# Assessing cross-resistance within the pyrethroids in terms of their interactions with key cytochrome P450 enzymes and resistance in vector populations

**DOI:** 10.1186/s13071-021-04609-5

**Published:** 2021-02-18

**Authors:** C. L. Moyes, R. S. Lees, C. Yunta, K. J. Walker, K. Hemmings, F. Oladepo, P. A. Hancock, D. Weetman, M. J. I. Paine, H. M. Ismail

**Affiliations:** 1 Big Data Institute, Li Ka Shing Centre for Health Information and Discovery, University of Oxford, Oxford, OX3 7LF UK; 2 Vector Biology Department, Liverpool School of Tropical Medicine, Pembroke Place, Liverpool, L3 5QA UK; 3 Institute of Life Course and Medical Sciences, University of Liverpool, Liverpool, L7 8TX UK

## Abstract

**Background:**

It is important to understand whether the potential impact of pyrethroid resistance on malaria control can be mitigated by switching between different pyrethroids or whether cross-resistance within this insecticide class precludes this approach.

**Methods:**

Here we assess the relationships among pyrethroids in terms of their binding affinity to, and depletion by, key cytochrome P450 enzymes (hereafter P450s) that are known to confer metabolic pyrethroid resistance in *Anopheles gambiae* (*s.l.*) and *An. funestus*, in order to identify which pyrethroids may diverge from the others in their vulnerability to resistance. We then investigate whether these same pyrethroids also diverge from the others in terms of resistance in vector populations.

**Results:**

We found that the type I and II pyrethroids permethrin and deltamethrin, respectively, are closely related in terms of binding affinity to key P450s, depletion by P450s and resistance within vector populations. Bifenthrin, which lacks the common structural moiety of most pyrethroids, diverged from the other pyrethroids tested in terms of both binding affinity to key P450s and depletion by P450s, but resistance to bifenthrin has rarely been tested in vector populations and was not analysed here. Etofenprox, which also lacks the common structural moiety of most pyrethroids, diverged from the more commonly deployed pyrethroids in terms of binding affinity to key P450s and resistance in vector populations, but did not diverge from these pyrethroids in terms of depletion by the P450s. The analysis of depletion by the P450s indicated that etofenprox may be more vulnerable to metabolic resistance mechanisms in vector populations. In addition, greater resistance to etofenprox was found across *Aedes aegypti* populations, but greater resistance to this compound was not found in any of the malaria vector species analysed. The results for pyrethroid depletion by anopheline P450s in the laboratory were largely not repeated in the findings for resistance in malaria vector populations.

**Conclusion:**

Importantly, the prevalence of resistance to the pyrethroids α-cypermethrin, cyfluthrin, deltamethrin, λ-cyhalothrin and permethrin was correlated across malaria vector populations, and switching between these compounds as a tool to mitigate against pyrethroid resistance is not advised without strong evidence supporting a true difference in resistance.
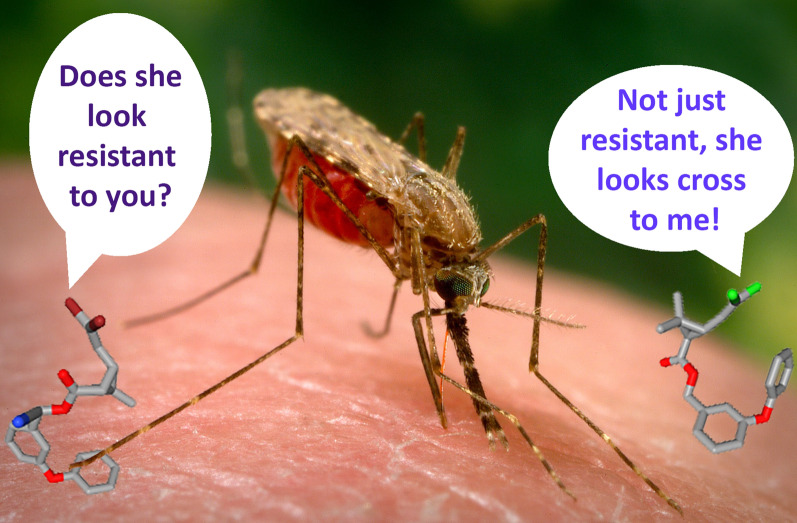

## Introduction

**Fig. 1:**
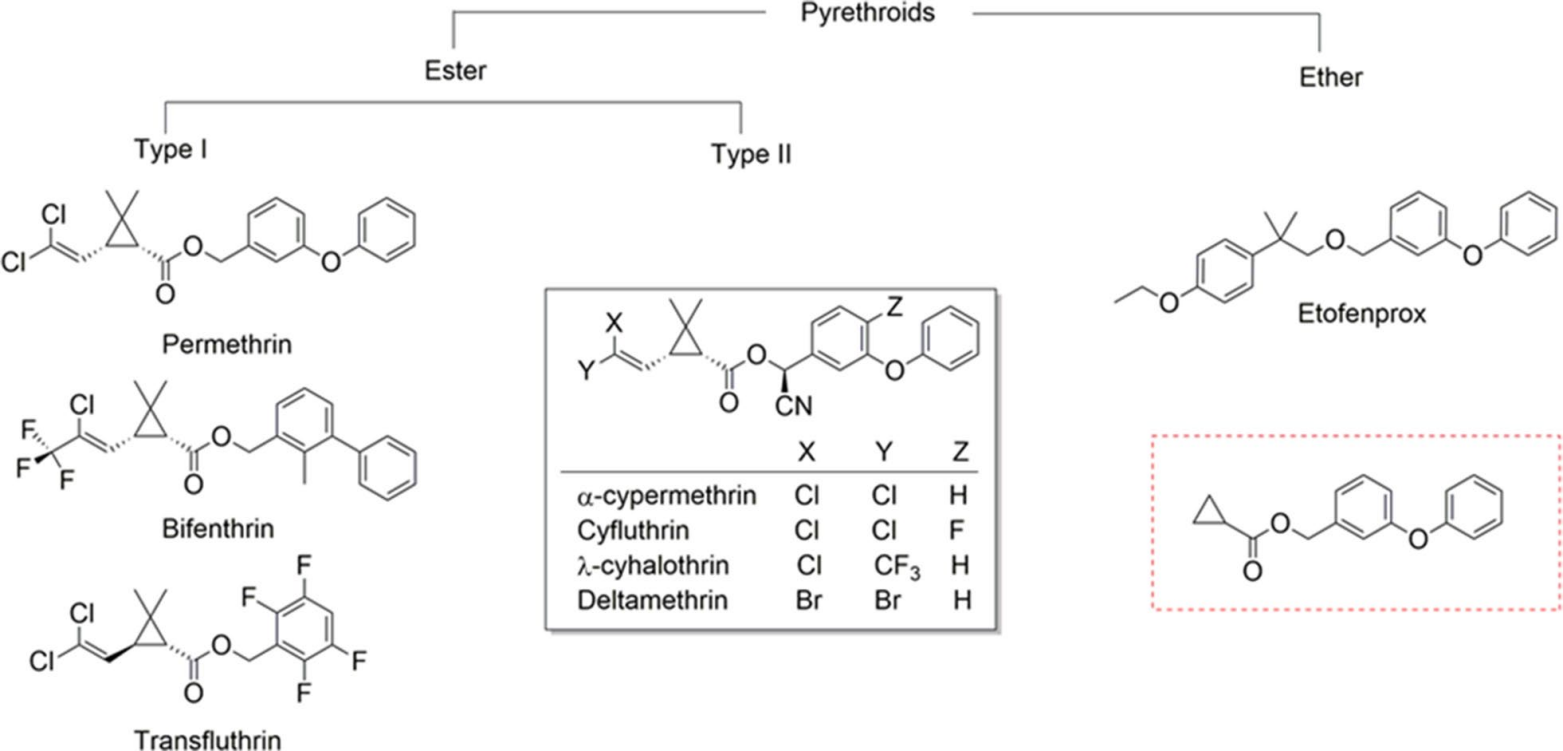
Chemical structure of pyrethroid insecticides used for malaria vector control. The common scaffold of pyrethroids, boxed in red, was identified by searching 230 million compounds available in the ZINC database (https://zinc.docking.org)

The primary malaria control intervention in high-burden countries is the deployment of long-lasting insecticide-treated nets (LLINs) impregnated with pyrethroids, alone or in combination with a second active ingredient or synergist [1, 2]. Widespread and increasing resistance to pyrethroids is, therefore, a serious potential threat to malaria control [3, 4]. Because the options for LLINs are limited, it is essential to understand whether the impact of resistance can be mitigated by switching between different pyrethroids or whether cross-resistance within this insecticide class precludes this approach. ‘Cross-resistance’ often refers to the instance where resistance is conferred to two or more classes of insecticide, is commonly assumed within an insecticide class. Evidence for divergence in resistance within an insecticide class may, however, be relevant especially given the reliance on a single insecticide class, the pyrethroids. Pyrethroids listed by the World Health Organization (WHO) for malaria control are differentiated into two groups based on biological activity that is associated with the absence (type I) or presence (type II) of an alpha-cyano group (Fig. 1). Type II pyrethroids are more lethal to insects because of their higher potency to the voltage-gated sodium channel (VGSC) in nerve membranes, the primary target site of pyrethroids [5, 6]. The higher potency of type II pyrethroids, such as deltamethrin and α-cypermethrin, translates into much lower doses being required to treat vector control products compared with type I pyrethroids, such as permethrin. This has led to increased deployment of alpha-cyano pyrethroids, in particular α-cypermethrin, which is currently used in 28% of the prequalified vector control products [2]. Generally, the pyrethroids used in vector control possess the common structural motif of phenoxybenzyl alcohol coupled with a cyclopropane ring via an ester bond, except for bifenthrin and etofenprox (Fig. 1). This narrow spectrum of chemical variation among pyrethroids makes it likely that cross-resistance will occur in malaria vector populations.

The high-burden countries where LLINs are deployed are concentrated in Africa where the most important vectors are *Anopheles gambiae* (*s.l.*) and *Anopheles funestus* [7]. Pyrethroid resistance in malaria vectors is primarily associated with target-site insensitivity due to mutations in the *Vgsc* gene known as knockdown resistance (*kdr*) and increased detoxification activity known as metabolic resistance. Metabolic mechanisms of resistance are found in all African malaria vectors, whereas *kdr* mutations are common in species of the *Anopheles gambiae* complex but not in the *An. funestus* subgroup [8–14]. There are multiple amino acid substitutions that cause target-site insensitivity resulting in pyrethroid resistance [15]. This includes a mutation, M918T, that produces a super-knockdown (*s-kdr*) phenotype in houseflies. Structure modelling studies in M918T phenotypes indicate that the highest degree of resistance in *s-kdr* houseflies depends on the chemical structure of the insecticide, which is positively correlated with the presence of an α-cyano group coupled with a phenoxybenzyl moiety in the larger type II pyrethroid molecules, such as deltamethrin and fenvalerate [16]. By comparison, the most common *Vgsc* resistance allele in west African *An. gambiae* populations*,* L1014F, is not influenced by pyrethroid chemical structure when expressed alone in house flies [17].

Although *kdr* mutations are common in *An. gambiae* (*s.l.*), they may have a relatively modest impact on resistance, and they are absent from highly pyrethroid-resistant *An. funestus* populations, suggesting that metabolic mechanisms may have a greater impact in African malaria vectors [18–20]. Metabolic resistance is most commonly mediated by elevated levels of cytochrome P450 (CYP) enzymes (hereafter referred to as P450s) [21]. Transcriptome-wide studies of gene expression in resistant and susceptible mosquito strains have found that upregulation of several cytochrome P450 genes is associated with resistance to both a type I pyrethroid (permethrin) and a type II pyrethroid (deltamethrin). For example, upregulation of the *CYP6P3* gene and its orthologues *CYP6P9a* and *CYP6P9b* and of the *CYP6AA1*, *CYP6Z1* and *CYP6Z3* genes is associated with resistance to both of these pyrethroids in *An*. *gambiae*/*An. coluzzii* and *An. funestus* [22–32]. In addition, upregulation of the *CYP6Z2* gene in *An. gambiae* and *An. coluzzii*, and of the *CYP6M7* gene in *An. funestus*, is also associated with resistance to both pyrethroids [23–25, 27, 28, 30–32]. These findings from studies of gene expression in resistant and susceptible strains provide evidence for P450-mediated pyrethroid cross-resistance in *Anopheles* populations, particularly to deltamethrin and permethrin; however, associations among resistance to more than one pyrethroid have not always been found, a limited range of pyrethroids has been tested and these studies do not give an indication of whether cross-resistance is stronger between some pyrethroids than others. Like the *Anopheles* vectors, target-site mutations and metabolic resistance are also thought to be the main resistance mechanisms in *Aedes* mosquitoes [33, 34].

An assessment of the impact of individual structural variation within the pyrethroid class on resistance in the field is required to inform the best use of different compounds. A previous study assessed resistance in malaria vector populations at more than 1000 sites in Africa and showed that when spatio-temporal trends were separated from noise in the susceptibility test data, strong associations among the resistance trends for three structurally similar pyrethroids (deltamethrin, ʎ-cyhalothrin and permethrin) were found [35]. The variance in the mean percent mortality values was 28 for the west Africa model and 23 for the east Africa model, reflecting the noisiness of the mortality data. This study also noted that the prevalence of resistance to permethrin was typically higher than that to deltamethrin; however, caution is needed when interpreting differences found using susceptibility test data because they may be due to real differences in the prevalence of resistance or differences in the calibration of the diagnostic dose, or both. Diagnostic doses currently recommended for use were calculated by doubling the dose of a compound which kills 100% of a susceptible strain of a species, or doubling the LC_99_ (lethal concentration that incurs 99% mortality) in this strain [36, 37]. A robust recommendation should be based on data from multiple strains in different testing centres, but where this is not possible doses may not be well calibrated between compounds. It is clear that differences in resistance between individual pyrethroids cannot be generally assumed, but it remains unclear whether meaningful differences can occur, particularly when a wider range of pyrethroid chemistries is considered.

In the study reported here, we took a new approach to assess variation in resistance among pyrethroids. We first assessed differences in pyrethroid chemistry that influence inhibition of the key enzymes that confer metabolic resistance in African malaria vectors, and the rate of depletion of each pyrethroid by these enzymes [38]. Of the primary resistance genes, the P450 superfamily is most frequently associated with metabolic resistance to pyrethroids in malaria vectors. Therefore, we assessed the relative differences among six pyrethroids in terms of their molecular interactions with P450s from the major African malaria vectors by constructing a P450s structure–activity relationship model (P450s-SAR). We focussed on α-cypermethrin, deltamethrin and permethrin as most relevant for recommendations regarding the current LLIN options. However, for broader future consideration, we included bifenthrin, etofenprox, cyfluthrin and λ-cyhalothrin, all structurally varied pyrethroids that are also in the WHO’s prequalified list for malaria vector control (Fig. 1) [2]. We then analysed resistance to these pyrethroids in multiple vector populations to determine whether the relative differences found by P450s-SAR studies translated into relative differences in resistance within wild populations. This was supplemented by an analysis of resistance in arbovirus vector populations. Finally, the resistance associations found across insecticide classes were also analysed in order to put the relationships found within the pyrethroids into the wider context of cross-resistance generally and to further investigate whether cross-resistance predicted by laboratory studies can be detected as general trends in the field data.

## Material and methods

In order to test whether relationships identified by SAR studies can be detected in the field, we constructed dendrograms for the hierarchical relationships between pyrethroids found by a series of molecular and field studies, and then compared the dendrograms obtained.

### Relationships among pyrethroids in terms of functional activity data

Cytochrome P450 inhibition assays using fluorogenic probe substrates have become commonplace in drug discovery screening cascades and are a rapid method of screening for insecticide interactions with mosquito P450s to predict insecticide binding, metabolism, cross-resistance and synergism [38–41]. In this study, the half maximal inhibitory concentration (IC_50_), which provides a value for inhibition of each P450 by each pyrethroid (also referred to as ‘binding affinity’), and the percentage depletion, which gives a value for metabolism of each pyrethroid by each P450 (also referred to as vulnerability to metabolic attack), were both included as parameters to establish a P450s structural activity relationship model. This model was used to understand both the chemistry of the pyrethroids and the interaction with mosquito P450s that function as monooxygenases in metabolic resistance, to predict cross-resistance liabilities in vivo. A low IC_50_ value indicates that the pyrethroid being assessed is a potent inhibitor that may be able to counter resistance mediated by P450s. A low percentage depletion indicates low metabolism of the pyrethroid, which means that it may be less vulnerable to resistance mediated by P450s.

The IC_50_ values for permethrin, etofenprox and bifenthrin (type I) and deltamethrin, λ-cyhalothrin and α-cypermethrin (type II) pyrethroids that were exposed to recombinant P450s from the *An. gambiae* Kisumu strain (CYP6Z2, =6M2, -6P2, -6P3 and -9J5) and the *An. funestus* FUMOZ strain (CYP6P9a) were extracted from two studies [38, 41]. In addition, inhibition activity data for these pyrethroids exposed to CYP6Z3 from the *An. gambiae* Kisumu strain were also generated (Additional file 1).

The values for percentage depletion (metabolism) of each pyrethroid by three of the enzymes CYP6M2, CYP6P3 and CYP6P9a, which were expressed in a single plasmid construct, were also extracted from the same sources and used for the comparative analysis.

The two datasets were analysed using hierarchical clustering of rows (insecticide) and columns (P450) by Perseus v1.6.14.0 to produce two visual heat maps representing the clustered matrices for relative insecticide binding affinity and insecticide vulnerability to metabolic attack. The clustered matrices for functional activity data for these six pyrethroids against these seven P450s were then used to construct dendrograms for the hierarchical relationships among the pyrethroids.

### Relationships among pyrethroids in terms of susceptibility test mortality in malaria vector populations

We accessed a published database of insecticide resistance in African malaria vectors [14] and identified all instances in which a mosquito sample from the field had been tested using two or more pyrethroids. Pairs of results were extracted, rather than instances in which a sample had been divided between tests of three or more pyrethroids, because there were insufficient data from studies testing > 2 pyrethroids against a single mosquito collection. Each data point provided paired susceptibility test data from a single collection sampled at a given time and place that was subdivided and subsequently tested under identical experimental conditions, with the aim of addressing the question ‘for a given time, place, species/complex and method, does higher resistance to pyrethroid A indicate higher resistance to pyrethroid B?’. A total of 3153 pairs of WHO susceptibility test results from samples of the *An*. *gambiae* complex were obtained. Only data that detected resistance to at least one pyrethroid were included; that is, results from samples that demonstrated 100% mortality to all of the pyrethroids tested were excluded.

We conducted a series of correlation analyses to assess how closely associated each pair of pyrethroids is in terms of resistance. The mean value for the Pearson’s correlation coefficient was calculated across 1000 bootstrapped samples for each pyrethroid pair using SPSS Statistics v25 (IBM Corp., Armonk, NY, USA). A Holm-Bonferroni correction was applied to identify significant correlations among the multiple tests conducted while avoiding false positives [42]. The mean correlation coefficients generated were ranked to identify the most and least closely correlated pyrethroids, respectively. These bootstrap mean correlation coefficients were used to construct a dendrogram of the hierarchical relationships among pyrethroids using the unweighted pair-group method with arithmetic mean [43], where the highest correlation coefficient indicated the most closely related pair.

The analyses conducted using data from *An. gambiae* (*s.l.*) samples were repeated using data from the *An. funestus* subgroup, *An. arabiensis*, *An. coluzzii*, *An. funestus* and *An. gambiae* samples (Additional file 2). The same approach was also used for susceptibility test data from *Aedes albopictus* and *Ae. aegypti* to investigate whether the same relationships could be detected in these vectors of arboviruses, as detailed in Additional file 3. There were much lower data volumes for the individual *Anopheles* species, compared to *An. gambiae* (*s.l.*), and a limited selection of pyrethroid pairs could be tested so no dendrograms were constructed from these data. Finally, the correlations between resistance to deltamethrin and resistance to insecticides from other classes were calculated in order to put the relationships found within the pyrethroids into the broader context of cross-resistance.

## Results

### Relationships among pyrethroids in terms of functional activity data

**Fig. 2:**
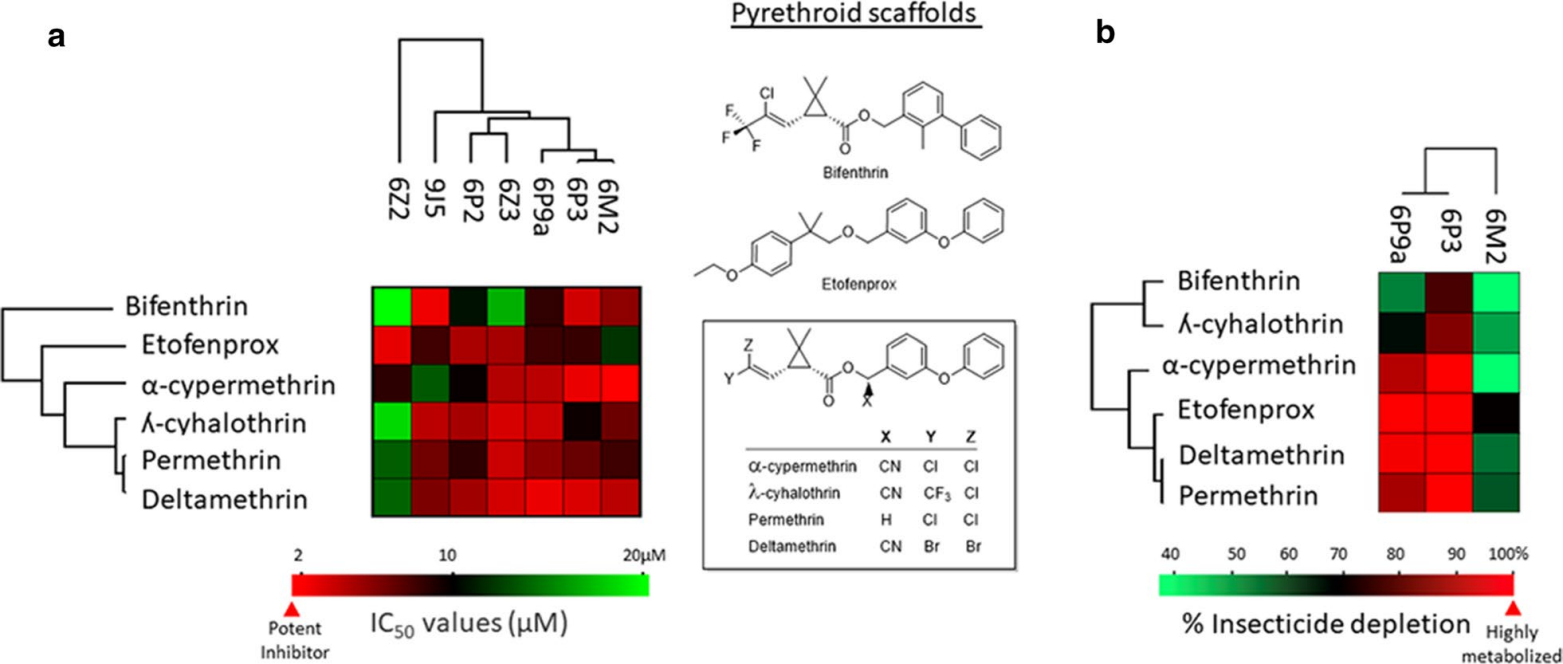
Cluster analysis of functional activity data for six pyrethroids against cytochrome P450 (CYP) enyzmes (P450s) from African malaria vectors. **a** Inhibition data from the screening of six pyrethroids (scaffold structures indicated on the right of data panels) against a set of P450s are presented as a heat map. Target enzymes are arrayed along the *x-axis*, and each of the pyrethroids is arrayed along the *y-axis*. Colours indicate the inhibition potency of pyrethroids with an indicated variable scaffold for a designated target P450. Potent (hot) inhibitors are assigned a red colour, and weak or ineffective (cold) inhibitors are given a light-green colour. **b** Pyrethroid metabolism by the P450s most widely associated with resistance from *Anopheles gambiae* (CYP6M2 and CYP6P3) and *An. funestus* (CYP6P9a) is clustered and presented as a heat map. Pyrethroids susceptible to metabolism are assigned a red colour, and weak metabolism is denoted light green. Dendrograms were obtained by hierarchical clustering and indicate the degree of similarity as a function of the height of the lines connecting the profiles

The six pyrethroids were categorised according to their inhibition of diethoxyfluorescein metabolism by P450s as potent (IC_50_ < 1 μM), moderate (IC_50_ 1–10 μM) and weak inhibitors (IC_50_ > 10 μM) [44]. Accordingly, all pyrethroids investigated showed low to moderate binding to the P450 panel (Fig. 2a; Additional file 1: Table S2). Bifenthrin had the lowest binding to the P450s panel examined (Fig. 2a; Additional file 1: Table S2).

CYP6P3, -6M2 and -6P9a were selected for comparative metabolism analysis because they are commonly associated with pyrethroid resistance, are among the earliest pyrethroid resistance markers to be functionally validated and are most heavily used for in vitro screening [29, 41, 45, 46]. All of the pyrethroids, with the exception of bifenthrin, were strongly metabolised by CYP6P3 and its orthologue CYP6P9a expressed from *An. gambiae* and *An. funestus*, respectively (Fig. 2b; Additional file 1: Table S3). However, lower metabolism profiles were observed with CYP6M2 expressed from *An. gambiae* (Fig. 2b; Additional file 1: Table S3). Notably, etofenprox was strongly metabolised by CYP6P3, CYP6M2 and CYP6P9a. Overall, the metabolism data presented in Fig. 2b and Additional file 1: Table S3 ranked etofenprox, deltamethrin and permethrin as the most vulnerable insecticides for metabolic attack by the three enzymes, followed by α-cypermethrin and ʎ-cyhalothrin; bifenthrin demonstrated the lowest vulnerability.

The dendrograms indicate that, in terms of inhibition of P450s and metabolism by P450s, permethrin and deltamethrin are closely related whereas bifenthrin diverges from these pyrethroids, (Fig. 2).

### Relationships among pyrethroids in terms of susceptibility test mortality in malaria vector populations

**Fig. 3:**
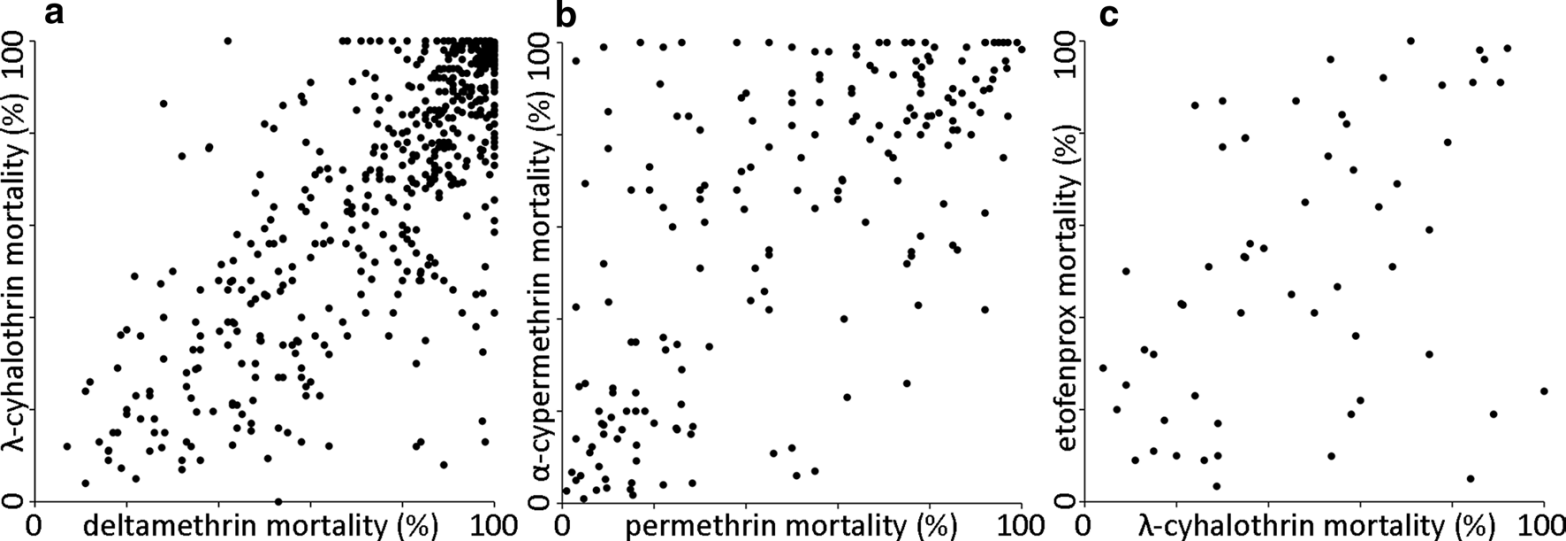
Distributions of values for three example pyrethroid pairs. **a** The most closely related pyrethroid pair in terms of resistance in wild mosquito populations (deltamethrin and λ-cyhalothrin), **b** a mid-ranked pyrethroid pair (permethrin and α-cypermethrin), **c** the least closely related pyrethroid pair (λ-cyhalothrin and etofenprox). Each point represents the results from a single *An. gambiae* (*s.l.*) sample that was subdivided between two susceptibility tests. The plots for all pairs are shown in Additional file 2: Figure S2

**Fig. 4:**
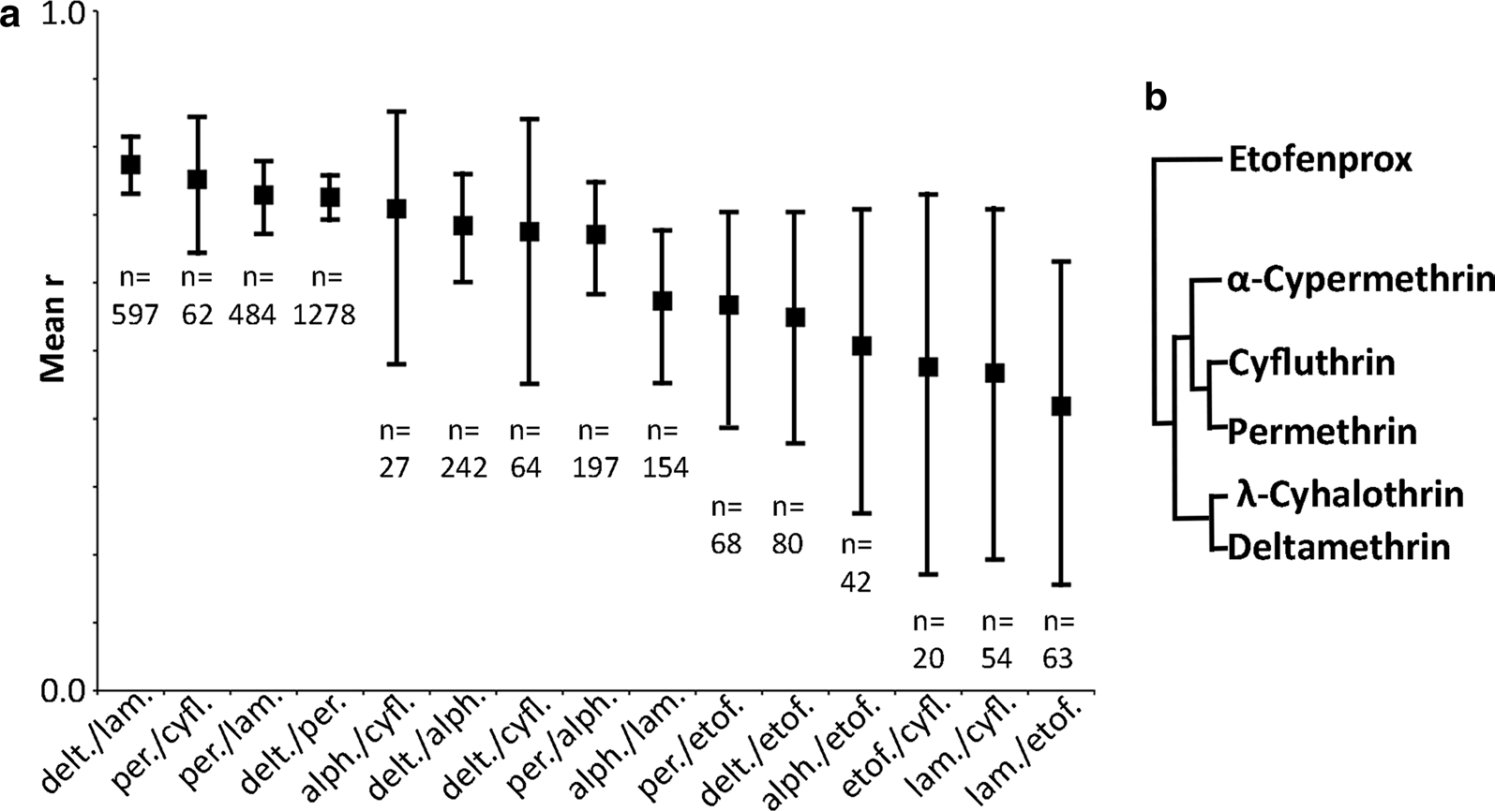
Relationships among pyrethroids defined by correlations in resistance within *An. gambiae* complex mosquitoes. **a** The mean correlation coefficient for each pyrethroid pair ranked from the most closely correlated to the most divergent.* alph.* α-Cypermethrin,* cyfl.* cyfluthrin,* delt.* deltamethrin,* etof.* etofenprox,* lamb.* λ-cyhalothrin,* per.* permethrin. The bars represent the upper and lower 95% bootstrap confidence interval and the sample size for each pair is given below these bars. **b** The hierarchical relationships among pyrethroids defined using the correlation coefficients shown in **a**

Each of the 15 pairs of values for pyrethroid resistance within *An. gambiae* (*s.l.*) was significantly correlated (Table 1). That is, populations with a higher prevalence of resistance to one pyrethroid tended to have a higher prevalence of resistance to the others (Fig. 3; Additional file 2: Figure S2). The pyrethroid pairs were ranked from the most closely correlated pair, deltamethrin* versus* λ-cyhalothrin, to the most divergent pair, etofenprox* versus* λ-cyhalothrin (Table 1; Fig. 4a). The correlation coefficients were used to construct a dendrogram of the hierarchical relationships among these pyrethroids (Fig. 4b). Deltamethrin, λ-cyhalothrin, permethrin, cyfluthrin and α-cypermethrin were closely related whereas etofenprox diverged from the other five pyrethroids.

**Table 1:** Correlations in resistance to seven pyrethroids in the *Anopheles gambiae* complex

Rank	Pyrethroid pair^a^	Paired sample size (*N*)	Mean* r*
1	Deltamethrin* vs* λ-cyhalothrin	597	0.774*
2	Permethrin* vs* cyfluthrin	62	0.752*
3	Permethrin * vs* λ-cyhalothrin	484	0.729*
4	Deltamethrin*vs* permethrin	1278	0.726*
5	α-Cypermethrin* vs* cyfluthrin	27	0.709*
6	Deltamethrin* vs* α-cypermethrin	242	0.684*
7	Deltamethrin* vs* cyfluthrin	64	0.675*
8	Permethrin* vs* α-cypermethrin	197	0.671*
9	λ-Cyhalothrin* vs* α-cypermethrin	154	0.573*
10	Permethrin* vs* etofenprox	68	0.567*
11	Deltamethrin* vs* etofenprox	80	0.549*
12	α-Cypermethrin* vs* etofenprox	42	0.507*
13	Etofenprox* vs* cyfluthrin	20	0.476*
14	λ-Cyhalothrin* vs* cyfluthrin	54	0.467*
15	λ-Cyhalothrin* vs* etofenprox	63	0.418*

*r*, Pearson’s correlation coefficient

*Significant results (at the 0.05 level with a Holm-Bonferroni correction)

^a^The most closely correlated pair is ranked first

### Comparison of pyrethroid relationships seen in the molecular and field studies

**Fig. 5:**
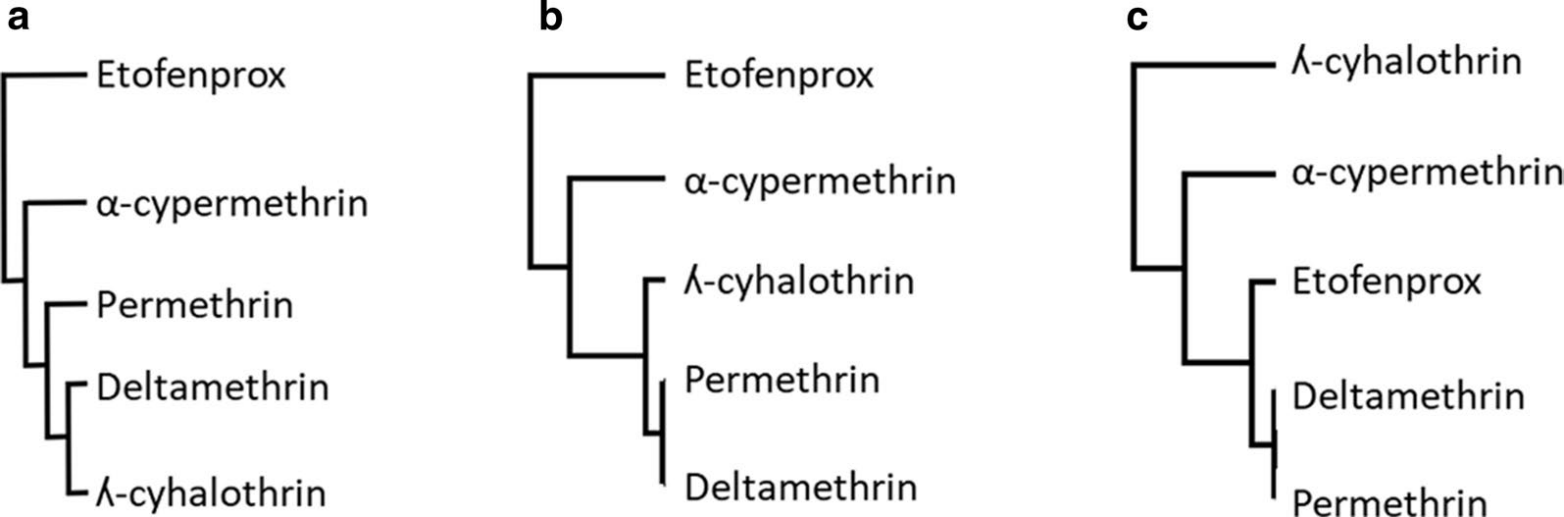
Hierarchical relationships among pyrethroids defined using data on resistance in vectors and functional activity data. The dendrograms were constructed using correlations in mortality across African malaria vector populations (Pearson’s correlation coefficient) (**a**), binding affinity values (IC_50_) (**b**) and insecticide depletion values (%) (**c**)

The three dendrograms using data for (i) resistance in field populations, (ii) P450 inhibition and (iii) depletion by P450s were re-constructed incorporating only the five pyrethroids that were included in all three analyses (Fig. 5). The dendrograms for P450 inhibition (also referred to as binding affinity) and vector population resistance both show that deltamethrin, λ-cyhalothrin, permethrin are most closely related to each other, followed by α-cypermethrin, with etofenprox as the most divergent (Fig. 5a, b). The dendrogram constructed using values for insecticide depletion (also referred to as vulnerability to metabolic attack) by CYP6P3, CYP6M2 and CYP6P9a reveals different relationships among these pyrethroids, although permethrin and deltamethrin are still closely related (Fig. 5c).

### Correlations in pyrethroid resistance within malaria vector species

Across *An. funestus* subgroup communities, there were significant correlations between resistance to deltamethrin and λ-cyhalothrin, permethrin and λ-cyhalothrin, and deltamethrin and permethrin, and the same was true for the four species tested (Table 2; Additional file 2: Figures S3 and S4). There were insufficient data to test the other pyrethroid combinations for the African malaria vector species. Across *Ae. aegypti* populations, resistance to cyfluthrin, deltamethrin, λ-cyhalothrin and permethrin was significantly correlated, whereas there were no significant correlations between these four pyrethroids and etofenprox (full results are given in Additional file 3).

**Table 2.** Correlations between resistance to different pyrethroids in African malaria vector species.

Pyrethroid combination	Paired sample size (*N*)	*r*
Deltamethrin* vs* λ-cyhalothrin		
* Anopheles funestus* subgroup	46	0.818*
* Anopheles funestus*	24	0.865*
* Anopheles arabiensis*	28	0.946*
* Anopheles coluzzii*	18	0.863*
* Anopheles coluzzii/gambiae*^a^	19	0.603*
* Anopheles gambiae*	19	0.418 n.s.
Permethrin* vs* λ-cyhalothrin		
* Anopheles funestus* subgroup	26	0.786*
* Anopheles funestus*	16	0.845*
* Anopheles arabiensis*	31	0.859*
* Anopheles coluzzii*	14	0.740*
* Anopheles coluzzii/gambiae*^a^	17	0.790*
* Anopheles gambiae*	4	Not tested
Deltamethrin* vs* permethrin		
* Anopheles funestus* subgroup	113	0.608*
* Anopheles funestus*	69	0.726*
* Anopheles arabiensis*	116	0.840*
* Anopheles coluzzii*	48	0.793*
* Anopheles coluzzii/gambiae*^a^	63	0.714*
* Anopheles gambiae*	75	0.782*

n.s., non-significant results (at the 0.05 level with a Holm-Bonferroni correction)

*Significant results (at the 0.05 level with a Holm-Bonferroni correction)

^a^*Anopheles coluzzii/gambiae* refers to mosquito samples that were undifferentiated between* An. coluzzii* (M form) and* An. gambiae* (S form), before they were recognised as two species

In order to put the relationships found within the pyrethroids into the wider context of cross-resistance across the insecticide classes used for malaria vector control, the correlations between deltamethrin and six commonly used non-pyrethroid insecticides were also calculated. Significant correlations with the prevalence of resistance to dichlorodiphenyltrichloroethane (DDT) were found for species within the *An. gambiae* complex but not for *An. funestus* (Additional file 2: Table S4, Figure S5). No significant correlations were found between the prevalence of resistance to deltamethrin and that to bendiocarb or propoxur (carbamates), malathion, fenitrothion or pirimiphos-methyl (organophosphates) for species within the *An. gambiae* complex or *An. funestus*.

### Variation in pyrethroid resistance within populations of African malaria vector species

The results presented above show significant correlations in resistance among the pyrethroids tested, but this result does not preclude the possibility that the prevalence of resistance is generally higher in one pyrethroid compared to the others across populations with differing levels of pyrethroid resistance. The insecticide depletion data presented above indicates that some pyrethroids are potentially more vulnerable to P450 attack; this is particularly the case for etofenprox which was most depleted by the three P450s (Additional file 1: Table S3). This leads to the question of whether higher levels of resistance to this compound can be detected in wild mosquito populations. An analysis of the paired data from *An. gambiae* (*s.l.*) samples collected across Africa provides no evidence that the prevalence of resistance is consistently higher for etofenprox compared to the other pyrethroids in *An. gambiae* (*s.l.*) (Additional file 2: Figure S6), but mortality was significantly lower after *Ae. aegypti* populations were exposed to etofenprox compared to mortality following exposure to deltamethrin, cyfluthrin, λ-cyhalothrin and permethrin (Additional file 3: Table S7).

To put the mortality differences found among pyrethroids (Additional file 2: Figures S7–S8) into the wider context of cross-resistance, the prevalence of resistance to deltamethrin was compared to the prevalence of resistance to six non-pyrethroid insecticides in paired susceptibility tests (Additional file 2: Figure S9). A reversal in the differences between resistance to deltamethrin and to the organochlorine DDT was found, with *An. gambiae* (*s.l.*) species having significantly higher resistance to DDT whereas *An. funestus* had significantly higher resistance to deltamethrin. In all species tested, mortality was lower following deltamethrin exposure compared to exposure to bendiocarb and propoxur (carbamates), malathion, fenitrothion and pirimiphos-methyl (organophosphates), respectively.

## Discussion

The results of this study highlight which of the pyrethroids used in malaria control are closely related in terms of inhibition of and depletion by P450s. Other studies of structurally diverse pyrethroids have also shown variation in P450 metabolism of pyrethroids with different structures. An in vivo study of the *An. funestus* strain, FUMOZ-R, which is characterised by upregulated P450 levels without any target-site mutations, found that transfluthrin, which contains a polyfluorobenzyl alcohol, was effective in the absence of the generic P450 inhibitor, piperonyl butoxide (PBO), whereas the other pyrethroids that contain the common phenoxybenzyl moiety, including cypermethrin, ß-cyfluthrin, deltamethrin and permethrin, were only effective when partnered with PBO [47]. This effect was associated with an inability of detoxifying enzymes to bind to the uncommon structure of transfluthrin. A similar observation was reported earlier from agriculture studies where an isogenic metabolic resistance strain isolated from a pyrethroid-resistant field population of *Helicoverpa armigera* showed significant cross-resistance between pyrethroids characterised by having both the phenoxybenzyl and aromatic acid moieties whereas the substitution of the phenoxybenzyl group with a polyfluorobenzyl group, as occurs in tefluthrin, benfluthrin and transfluthrin, overcame most of this resistance [48]. These studies support the aim of identifying pyrethroids that are active against resistant populations when P450-mediated resistance plays a major role. In our study, bifenthrin diverged from the other pyrethroids in terms of both inhibition of, and depletion by, P450s, but no susceptibility test data were available for resistance to bifenthrin in populations of African malaria vectors. Susceptibility test data were available for etofenprox, and this pyrethroid was found to diverge from the more commonly deployed pyrethroids in terms of inhibition of *An. gambiae* and *An. funestus* P450s and in terms of resistance in *An. gambiae* (*s.l.*) and *Ae. aegypti* populations.

The susceptibility test data from these populations show strong associations between resistance to the most commonly used pyrethroids (deltamethrin, λ-cyhalothrin, permethrin and α-cypermethrin), in agreement with the results for binding affinity and with earlier studies of spatio-temporal trends in *An. gambiae* (*s.l.*) [3, 35]. The correlations in resistance among these pyrethroids, which were demonstrated in all the major African malaria vectors, suggest that if differences in resistance to these pyrethroids (as well as to the less commonly deployed cyfluthrin) are found using susceptibility tests conducted on a small number of field samples of malaria vectors, further evidence should be obtained before any decision is made to switch between them.

Greater differentiation was found for resistance to bifenthrin in terms of both inhibition of, and depletion by, P450s. The results for bifenthrin are interesting because they show that (i) this pyrethroid differs from the other pyrethroids in terms of P450 binding and metabolism and that (ii) it may be less susceptible to common P450 enzymes. Bifenthrin is the active ingredient in one indoor residual spray (IRS), Bistar 10WP [2, 49], which is used in India. Bifenthrin IRS was trialled in Nigeria in 2006 and Zambia in 2011 [50–52], but it has not been widely deployed in Africa where concerns about the duration of residual activity have been raised [52–54]. There are no field data from susceptibility tests on African malaria vectors conducted using bifenthrin, presumably because this compound is rarely deployed and because there is no recommended diagnostic dose for use in a susceptibility test. One study of *Anopheles sinensis* in Korea collected blood-fed adults in the field and exposed subsets of the F1 larvae to each of the pyrethroids considered in our study. Resistance ratios using LC_50_ values were calculated from a susceptible strain, and the results revealed that the larvae were most susceptible to bifenthrin, cyfluthrin and etofenprox, in that order, and least susceptible to permethrin [55]. Further evidence comes from studies of *Aedes* vectors, including three studies that tested bifenthrin [34]. One study in Mexico tested seven populations of *Aedes aegypti* with eight pyrethroids and compared the concentrations required for 50% knockdown (KC_50_) and mortality (LC_50_) to the same values obtained using a susceptible strain to give a resistance ratio (RR) [56]. Across the seven populations, resistance to deltamethrin, lambda-cyhalothrin, permethrin and α-cypermethrin were highly correlated (in terms of both RRKC_50_ and RRLC_50_), indicating the existence of strong cross-resistance. However, the resistance values for bifenthrin were not correlated with any of those for the other four compounds, and the authors of the study concluded that bifenthrin could be an alternative insecticide for *Ae. aegypti* in Mexico. Two independent studies in Thailand tested three *Ae. aegypti* and three *Ae. albopictus* populations, respectively, and calculated the diagnostic doses for each pyrethroid, including bifenthrin, using a susceptible strain [57, 58]. In both studies, the population with the highest deltamethrin resistance also had the highest bifenthrin resistance, so no evidence for divergence in resistance was observed for these two species in Thailand. Given the known data noise in susceptibility test results, caution is needed when interpreting the results from a single study at a small number of sites. It is also worth noting that bifenthrin’s relative immunity to depletion by CYP6M2, CYP6P3 and CYP6P9a described here was not found when tested previously [28]. Metabolism assays carried out in two earlier studies showed that CYP6M7, CYP6P9a and CYP6P9b from *An. funestus* metabolised bifenthrin (62, 68 and 71%, respectively) as well as permethrin, deltamethrin and λ-cyhalothrin (ranging from 46 to 81% depletion). Field tests for bifenthrin resistance in malaria vector populations are needed before a firm conclusion can be reached on whether bifenthrin can be recommended in situations where resistance to other pyrethroids has been found.

The analyses of binding affinity data and of field data from malaria vector populations both show that resistance to etofenprox diverges, to a degree, from resistance to the more commonly deployed pyrethroids. This result is backed up by data from studies of resistance in *Ae. aegypti*. However, the depletion activity data suggest that etofenprox is more vulnerable to P450 metabolism and that if resistance to this compound is found to be greater in malaria vector populations, then a switch would not be advised. A trend for higher resistance to etofenprox was not seen in the data from malaria vector populations but it was found in the data from *Ae. aegypti* populations, although caution is needed when interpreting differences found using susceptibility test data (particularly tests using diagnostic doses that have not been calibrated for *Aedes* species [34]). Etofenprox is the active ingredient in two WHO prequalified products, namely a kit for insecticide-treated nets (Vectron 10EW) and an IRS formulation (Vectron20WP) [2]. The latter product is listed by the Global Fund, but etofenprox is not widely deployed in Africa and was last reported as the active ingredient used for IRS in 2012 in parts of Zambia [51, 52].

We found some variation in the relationships among pyrethroids when different types of evidence were considered. In particular, the results for insecticide depletion were largely not repeated in the findings for resistance in mosquito populations. The results for both insecticide inhibition and insecticide depletion depend on which enzymes are included in the activity tests. Seven P450s (three for the depletion analysis) were included in the present study, whereas at least 14 have been implicated in *An. gambiae* (*s.l.*) and *An. funestus* resistance to date [21, 22, 24–32, 46, 59–73] and many more in* Aedes* vectors [34]. It is also important to note that detoxification by P450s is not the only mechanism of resistance found in these vector species. Target-site mutations are common in many of these species [9–13], upregulation of other detoxifying enzymes is also linked to pyrethroid resistance [74] and there is some evidence for cuticular thickening in resistant mosquitoes [75]. Upregulation of the *GSTE2* gene is associated with resistance to both permethrin and deltamethrin, as well as DDT, in *An. gambiae* and *An. coluzzii* [70, 72, 76], *An. funestus* [29, 71, 74] and *Ae. aegypti* [77–79], and allele frequencies for target-site mutations in the voltage-gated sodium channel gene, *Vgsc*, have been shown to be useful partial predictors of resistance in *An. gambiae* (*s.l.*) [35]. Thus, we would not expect the findings from molecular studies of P450 activity alone to be exactly replicated in field populations, except in instances where P450-mediated metabolic resistance dominates in a mosquito population.

The results for pyrethroid cross-resistance within individual species reported here match our knowledge of other mechanisms of resistance found in these species. Mutations in the *Vgsc* gene (*kdr* mutations) confer cross-resistance to pyrethroids and DDT, and are partial predictors of patterns of resistance to these compounds in the *An. gambiae* complex, but they have not been found in *An. funestus* or other members of the *An. funestus* subgroup [3, 8–14, 35]. In our study, correlations between pyrethroid and DDT resistance were found for members of the *An. gambiae* complex but not for the *An. funestus* subgroup or species. No correlations were found between pyrethroid resistance and resistance to the carbamates or organochlorines, underlining the finding that it is cross-resistance within the pyrethroids, as well as between the pyrethroids and DDT, that is most important. Some metabolic resistance mechanisms do confer cross-class resistance, for example between the pyrethroids and DDT and/or the carbamates [24, 30, 32, 73], but the impact of these mechanisms within the array of resistance types that co-occur is more nuanced, and no cross-class resistance other than the aforementioned pyrethroid–DDT resistance in *An. gambiae* (*s.l.*) was detected here.

In conclusion, we have shown that the type I and type II pyrethroids permethrin and deltamethrin, respectively, are closely related, as exemplified by (i) the close associations between the binding affinities of permethrin and deltamethrin to a range of anopheline P450s, (ii) the close associations between depletion of permethrin and deltamethrin by these P450s and (iii) correlations in resistance to permethrin and deltamethrin in populations of *An. arabiensis*, *An. coluzzii*, *An. gambiae* and *An. funestus*. Importantly, a population with higher resistance to one of the pyrethroids incorporating the common structural motif of phenoxybenzyl alcohol coupled with a cyclopropane ring (the primary target for metabolic oxidation) is likely to have higher resistance to the others, and these cross-resistance trends could be detected despite the noise in the susceptibility test data. It is unlikely that resistance to those pyrethroids most commonly deployed for malaria control diverges within vector populations, and it would be unwise to switch between these compounds based on the results from a small number of susceptibility tests alone. There are, however, pyrethroids that are not commonly deployed that show greater potential for true divergence in resistance, such as bifenthrin and possibly etofenprox. Bifenthrin diverged from the other pyrethroids tested in terms of both binding affinity to key P450s and depletion by P450s, but resistance to bifenthrin has rarely been tested in vector populations and was not analysed here. Etofenprox diverged from the more commonly deployed pyrethroids in terms of binding affinity to key P450s and resistance in vector populations, but was closely related to these pyrethroids in terms of depletion by anopheline P450s in the laboratory. The analysis of pyrethroid depletion by the P450s indicates that etofenprox may be particularly vulnerable to metabolic resistance mechanisms in vector populations. In addition, greater resistance was found across *Ae. aegypti* populations, but greater resistance to etofenprox was not found in any of the malaria vector species analysed. It is worth noting that there are still significant correlations between resistance in malaria vector populations to etofenprox and resistance to the pyrethroids in common use, and it is possible that a correlation could also be found for bifenthrin once data from multiple vector populations are available to answer this question. Systematic SAR analyses of these more structurally diverse pyrethroids are required to estimate the effect of structural diversity on pyrethroid resistance, and these findings need to be verified by studies of resistance in wild populations.

## Supplementary Information

**Additional file 1.** Further details of the functional activity data including **Table S1.** Primers used for amplification of CYP6Z3 and in vitro functional characterisation. **Figure S1.** Fe2+-CO vs. Fe2+ difference spectrum of *E. coli* membranes expressing AgCYP6Z3. **Table S2.** IC50 values (μM) of pyrethroid insecticides. **Table S3**. Pyrethroid metabolism by mosquito P450s.

**Additional file 2.** Additional results from the population resistance comparisons including **Figure S2.** The distributions of paired pyrethroid susceptibility test mortality values for *An. gambiae* s.l. **Figure S3.** The distributions of paired pyrethroid susceptibility test mortality values for the *An. funestus* subgroup. **Figure S4.** The distributions of paired pyrethroid susceptibility test mortality values for four species. **Figure S5.** The distributions of paired susceptibility test mortality values for four species exposed to deltamethrin and a non-pyrethroid insecticide. **Figure S6.** Comparisons between resistance to etofenprox and five other pyrethroids in the *An. gambiae* complex and the *An. funestus* subgroup. **Figure S7.** Comparison of resistance to three pyrethroids within the *An. gambiae* complex and *An. funestus* subgroup. **Figure S8.** Comparisons of resistance to three pyrethroids in four malaria vector species. **Figure S9.** Comparison of resistance to deltamethrin and six insecticides from other classes. **Table S4.** Correlations between resistance to deltamethrin and non-pyrethroid insecticides in four African malaria vector species. 

**Additional file 3.** Further details of the analyses of resistance in *Aedes* populations including Table S5. Diagnostic doses and data volumes for each pyrethroid. Table S6. Correlations in resistance to different pyrethroids in *Ae. aegypti* samples. Table S7. Comparisons of mean mortality between pairs of pyrethroids.

**Additional file 4.** A csv file containing the full data for pairs of pyrethoid susceptibility test results in each species.

## Data Availability

All susceptibility test data analysed during this study are included in Additional file 4.
