# Supplementary material for: Assessing cross-resistance within the pyrethroids in terms of their interactions with key cytochrome P450 enzymes and resistance in vector populations
Source: Parasit Vectors. 2021 Feb 18;14:115. doi: 10.1186/s13071-021-04609-5 (PMC7893915; doi:10.1186/s13071-021-04609-5)
Supplement: Supplementary file 1 — Additional file 1. Further details of the functional activity data including Table S1. Primers used for amplification of CYP6Z3 and in vitro functional characterisation. Figure S1. Fe2+-CO vs. Fe2+ difference spectrum of E. coli membranes expressing AgCYP6Z3. Table S2. IC50 values (μM) of pyrethroid insecticides. Table S3. Pyrethroid metabolism by mosquito P450s. [file 13071_2021_4609_MOESM1_ESM.pdf]

Additional file 1

1. Preparation of *An. gambiae* CYP6Z3 and cloning

Total RNA was extracted with Arcturus PicoPure Kit (Applied Biosystems, USA) from ten adult *An. gambiae* mosquitoes from the Kisumu strain [1]. Complementary DNA was prepared using Superscript III (Invitrogen) with an oligo $(dT)_{20}$ primer and used as a template for amplifying full-length *AgCyp6z3* with KOD DNA polymerase (Merk Chemicals). The gene-specific primers used in these high-fidelity PCRs (Table S1) were designed according to the reference *An. gambiae* PEST CYP6Z3 genome sequence (Accession number AY193727) The PCR product was ligated into pGEM T-easy vector (Promega) and sequenced on both strands. Consistent with the high frequency of polymorphism in *An. gambiae* P450s [2](Martin Nature Ref) there were 36 single nucleotide polymorphism (SNPs) compared with the reference sequence, of which three produced amino acid changes, S209G, W396L, and H411Y. For expression, the ompA leader sequence (ompA) was engineered onto the amino-terminus of the *AgCyp6z3* gene to direct the P450 into the *E. coli* outer membrane during expression as previously described [3, 4]. The ompA-leader was fused to *AgCyp6z3* cDNA in frame with *AgCyp6z3* initiation codon by fusion PCR using High fidelity Phusion polymerase (Scientific). The ompA *AgCyp6z3* fusion was flanked at the 5′ and 3′ ends with NdeI and EcoR1, respectively for ligation into NdeI/EcoRI linearised pB13 (pCWori+).

Table S1. Primers used for amplification of CYP6Z3 and *in vitro* functional characterisation

Primer	Sequence (5′-3′)
CYP6Z3_forward	ATGGCTGTTTACACTCTCGCGCTCGT
CYP6Z3_reverse	TCGAATTCTCAGCATCTATGTTCTACGCGC
ompA+2 Forward	GGAATTCCATATGAAAAAGACAGCTATCGCG
ompA+2 CYP6Z3 fusion	CGCCACGAGCGCGAGAGTGTAAACAGCCATCGGAGCGGCCTGCG CTACGGTAGCGAA

2. Preparation of membranes expressing P450 and AgCPR

To express functional P450, *AgCyp6z3* cloned into pB13 plasmid was co-transformed into *E. coli* JM109 cells with *An. gambiae* NADPH-cytochrome P450 reductase (*AgCPR-pACYC*). The P450 and the *AgCPR* were previously fused with ompA and pelB leader sequences respectively to direct the expressed proteins to the inner bacterial periplasm in order to form functional monooxygenase complex [3, 5, 6]. For P450 production, 200 mL *E. coli* cultures in Terrific Broth were incubated at 21°C with shaking at 120 RPM for 18–24 h after induction with 1 mM IPTG. Following expression, *E. coli* membranes containing CYP6Z3 were isolated and P450 and AgCPR content measured as previously described [3, 7]. Samples were stored in aliquots of 100 µL at −80°C. *An. gambiae* cytochrome *b*5 was prepared as described previously to supplement enzyme reactions at an 8:1 M ratio, *b*5: P450 [4].

3. Inhibition screening of pyrethroids.

Inhibition screening of pyrethroid inhibitors and IC_{50} calculations carried out according Yunta et al. 2016[8]. Variable ligand concentrations were used for IC50 calculations with diethoxy fluorescein [DEF] used at ~Km (0.2 and 0.5 µM) with 0.1 µM of CYP6Z3 and CYP6Z2 respectively. DEF reactions (200 µL) were carried out in at 25 °C in 50 mM KPi at pH 7.4 containing 1 mM glucose-6-phosphate (G6P), 0.1 mM NADP+, 0.25 mM MgCl2, and cytochrome b5 at an 8:1 molar ratio, b5: P450. NADP+ and G6P were excluded from the minus NADPH controls. Three replicates of positive and negative control reactions were run for each P450/substrate combination in opaque white 96-well (flat-based) plates in triplicate. The IC_{50} fitting calculations were performed using GraphPad Prism 6. Data were fitted to the dose-response model and plots with R^2 <0.95 were rejected.

Results

The co-expression of *AgCYP6Z3* (AGAP008217) and *An. gambiae* NADPH-cytochrome P450 reductase (*AgCPR*) (AGAP000500) produced 8.25 µM P450 as measured by Fe2+-CO vs Fe2+ difference spectra [9] in membranes (Fig S1). The P450 reductase activity was in the range of 18.04 – 29.47 nmol/min/mg protein.

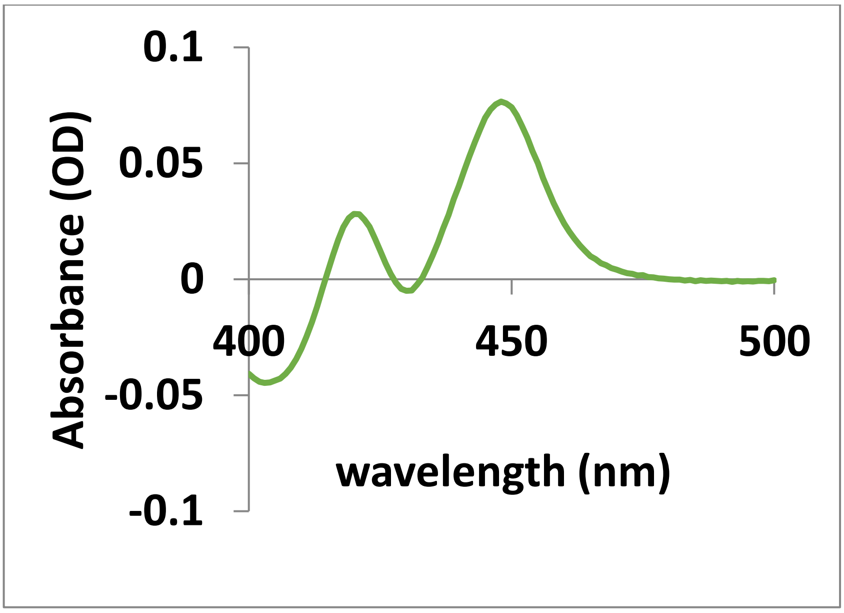

Fig. S1. Fe^{2+}-CO vs. Fe^{2+} difference spectrum of *E. coli* membranes expressing AgCYP6Z3.

Table S2. IC_{50} values (µM) of pyrethroids insecticides

Insecticide	CYP6M2	CYP6P3	CYP6P9a	CYP6P2	CYP9J5	CYP6Z3	CYP6Z2	Mean across P450s
Deltamethrin	4.2[a]	3.2[a]	2.6[a]	5.0[a]	6.1[a]	3.4	13.9[a]	5.5
α-cypermethrin	1.5[b]	2.6[b]	4.2[b]	9.8[b]	13.5[b]	4.4	8.6	6.4
Etofenprox	12[b]	8.3[b]	8.1[b]	4.7[b]	8[b]	4.8	2.8	7.0
λ-Cyhalothrin	6.6[b]	9.6[b]	3.7[b]	4.8[b]	4.2[b]	3.3	18.6	7.2
Permethrin	8.1[a]	6.8[a]	5.7[a]	8.6[a]	6.5[a]	3.7	13.7[a]	7.6
Bifenthrin	5.6[b]	3.5[b]	8.5[b]	10.7[b]	2.5[b]	17	20.7	9.8

[a] Published in Yunta *et al.* 2016[8] and [b] published in Yunta *et al*. 2019[10].

Table S3. Pyrethroid metabolism by mosquito P450s

P450	% Insecticide depletion					
	Pyrethroids type I			Pyrethroids type II		
	Permethrin	Etofenprox	Bifenthrin	Deltamethrin	λ-cyhalothrin	Cypermethrin
CYP6M2[t]	58.5 ± 2.2	68.8 ± 1.1	38.9 ± 1.6	55.4 ± 1.4	49.4 ± 0.5	36.8 ± 1.8
CYP6P3[t]	100.0 ± 0.0	99.8 ± 0.3	76.7 ± 0.3	98.2 ± 0.2	83.3 ± 15.4	98.4 ± 0.1
CYP6P9a[t]	87.8 ± 0.7	98.5 ± 0.2	53.4 ± 1.6	97.0 ± 0.2	67.1 ± 3.1	89.5 ± 2.7
Mean across P450s	82.1 ± 21.3	89.0 ± 17.5	56.3 ± 19.1	83.5 ± 24.2	66.6 ± 17.0	74.9 ± 33.3

t: Incubation time 2h; (mean ± SD)

References

1. Witzig, C., et al., *Genetic mapping identifies a major locus spanning P450 clusters associated with pyrethroid resistance in kdr-free Anopheles arabiensis from Chad.* Heredity, 2013. **110**(4): p. 389-397.
2. Miles, A., et al., *Genetic diversity of the African malaria vector Anopheles gambiae.* Nature, 2017. **552**(7683): p. 96-+.
3. McLaughlin, L., et al., *Characterization of inhibitors and substrates of Anopheles gambiae CYP6Z2.* Insect molecular biology, 2008. **17**(2): p. 125-135.
4. Stevenson, B.J., et al., *Cytochrome P450 6M2 from the malaria vector Anopheles gambiae metabolizes pyrethroids: Sequential metabolism of deltamethrin revealed.* Insect biochemistry and molecular biology, 2011. **41**(7): p. 492-502.
5. Pritchard, M.P., et al., *Functional co-expression of CYP2D6 and human NADPHcytochrome P450 reductase in Escherichia coli.* Pharmacogenetics and Genomics, 1998. **8**(1): p. 33-42.
6. Pritchard, M.P., et al., *A General Strategy for the Expression of Recombinant Human Cytochrome P450s inEscherichia coliUsing Bacterial Signal Peptides: Expression of CYP3A4, CYP2A6, and CYP2E1.* Archives of biochemistry and biophysics, 1997. **345**(2): p. 342-354.
7. Stevenson, B.J., et al., *Pinpointing P450s associated with pyrethroid metabolism in the dengue vector, Aedes aegypti: developing new tools to combat insecticide resistance.* PLoS neglected tropical diseases, 2012. **6**(3): p. e1595.
8. Yunta, C., et al., *Pyriproxyfen is metabolized by P450s associated with pyrethroid resistance in An. gambiae.* Insect Biochemistry and Molecular Biology, 2016. **78**: p. 50-57.

9. Omura, T. and R. Sato, *The Carbon Monoxide-Binding Pigment of Liver Microsomes. Ii. Solubilization, Purification, and Properties.* J Biol Chem, 1964. **239**: p. 2379-85.
10. Yunta, C., et al., *Cross-resistance profiles of malaria mosquito P450s associated with pyrethroid resistance against WHO insecticides.* Pesticide Biochemistry and Physiology, 2019. **161**: p. 61-67.
