# Supplementary material for: Assessing cross-resistance within the pyrethroids in terms of their interactions with key cytochrome P450 enzymes and resistance in vector populations
Source: Parasit Vectors. 2021 Feb 18;14:115. doi: 10.1186/s13071-021-04609-5 (PMC7893915; doi:10.1186/s13071-021-04609-5)
Supplement: Supplementary file 2 — Additional file 2. Additional results from the population resistance comparisons including Figure S2. The distributions of paired pyrethroid susceptibility test mortality values for An. gambiae s.l. Figure S3. The distributions of paired pyrethroid susceptibility test mortality values for the An. funestus subgroup. Figure S4. The distributions of paired pyrethroid susceptibility test mortality values for four species. Figure S5. The distributions of paired susceptibility test mortality values for four species exposed to deltamethrin and a non-pyrethroid insecticide. Figure S6. Comparisons between resistance to etofenprox and five other pyrethroids in the An. gambiae complex and the An. funestus subgroup. Figure S7. Comparison of resistance to three pyrethroids within the An. gambiae complex and An. funestus subgroup. Figure S8. Comparisons of resistance to three pyrethroids in four malaria vector species. Figure S9. Comparison of resistance to deltamethrin and six insecticides from other classes. Table S4. Correlations between resistance to deltamethrin and non-pyrethroid insecticides in four African malaria vector species. [file 13071_2021_4609_MOESM2_ESM.pdf]

Additional File 2. Further results for correlations found in susceptibility test results from wild vector populations

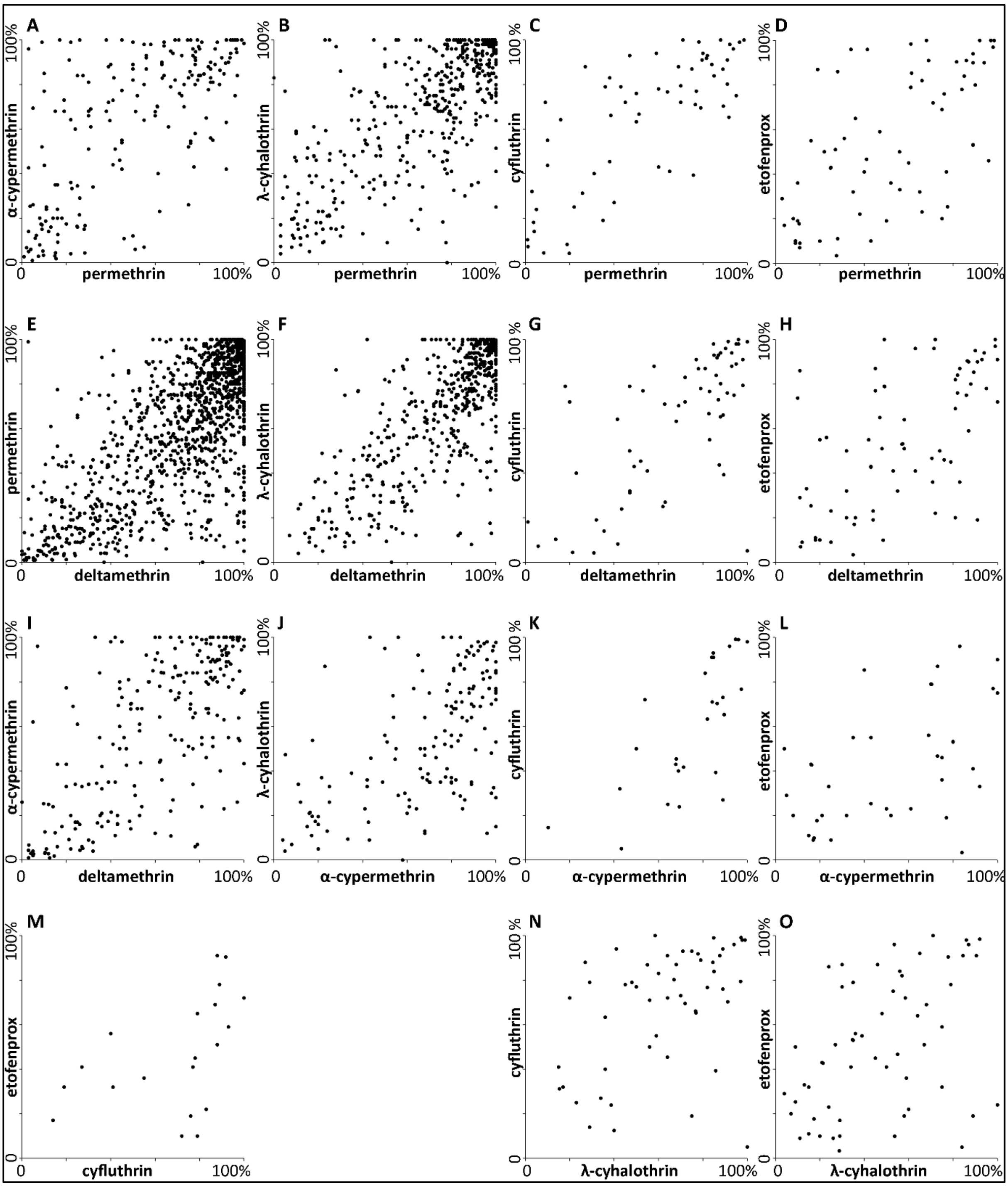

Figure S2. The distributions of paired pyrethroid susceptibility test mortality values for *An. gambiae* s.l. Each point represents the results from a single *An. gambiae* s.l. sample that was subdivided between susceptibility tests for two different pyrethroids to give a pair of percent mortality values.

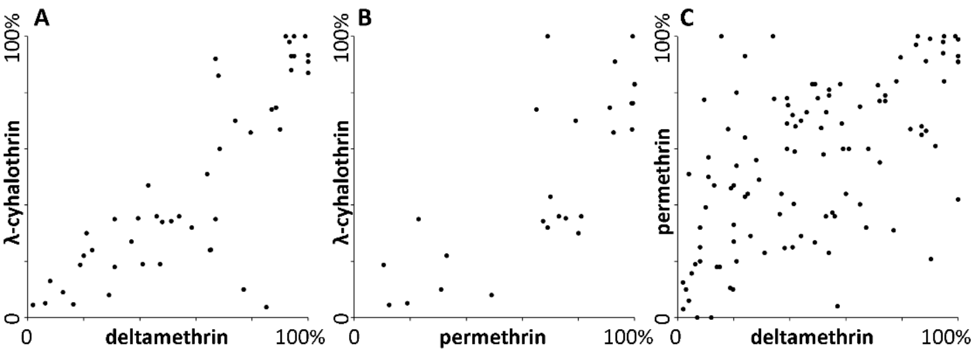

Figure S3. The distributions of paired pyrethroid susceptibility test mortality values for the *An. funestus* subgroup.

Figure S4. The distributions of paired pyrethroid susceptibility test mortality values for four species. Each point represents the results for a single species from a mosquito sample that was subdivided between susceptibility tests for two different pyrethroids to give a pair of percent mortality values.

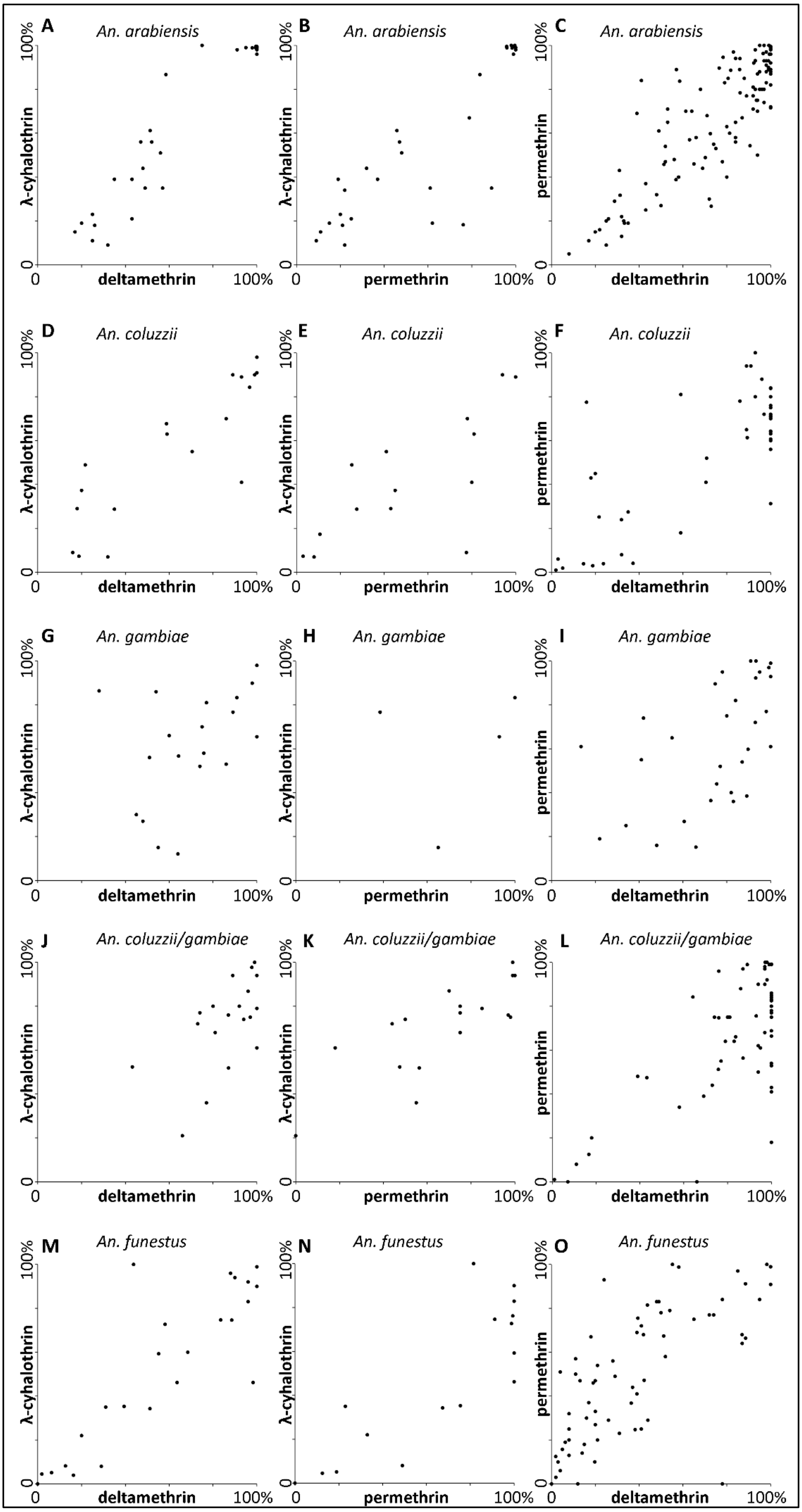

Figure S5. **The distributions of paired susceptibility test mortality values for four species exposed to deltamethrin and a non-pyrethroid insecticide**. Each point represents the results for a single species from a mosquito sample that was subdivided between two susceptibility tests for deltamethrin and a non-pyrethroid insecticide to give a pair of percent mortality values.

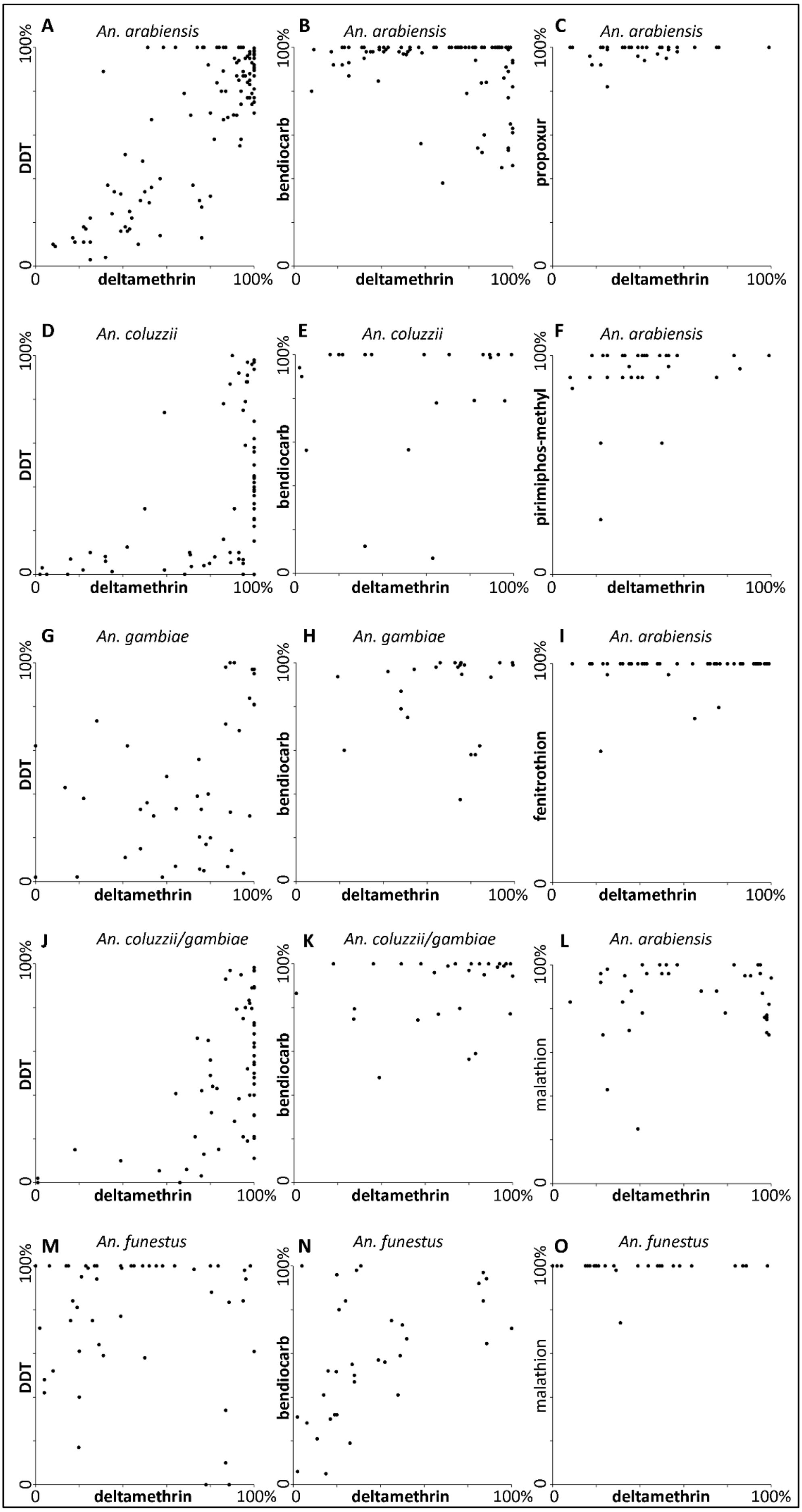

Table S4. Correlations between resistance to deltamethrin and non-pyrethroid insecticides in four African malaria vector species

**Deltamethrin vs DDT**	**N**	**Correlation**
Anopheles arabiensis	107	0.808*
Anopheles coluzzii	69	0.506*
Anopheles coluzzii/gambiae	62	0.545*
Anopheles gambiae	43	0.370 n.s.
Anopheles funestus	48	-0.071 n.s.
**Deltamethrin vs bendiocarb**		
Anopheles arabiensis	97	-0.224 n.s.
Anopheles coluzzii	22	0.133 n.s.
Anopheles coluzzii/gambiae	28	0.218 n.s.
Anopheles gambiae	22	0.073 n.s.
Anopheles funestus	35	0.439 n.s.
**Deltamethrin vs fenitrothion**		
Anopheles arabiensis	55	0.170 n.s.
**Deltamethrin vs malathion**		
Anopheles arabiensis	37	0.084 n.s.
Anopheles funestus	29	0.061 n.s.
**Deltamethrin vs pirimiphos-methyl**		
Anopheles arabiensis	34	0.246 n.s.
**Deltamethrin vs propoxur**		
Anopheles arabiensis	34	0.249 n.s.

Significant results (at the 0.05 level with a Holm-Bonferroni correction) are denoted by *. The term 'Anopheles coluzzii/gambiae' refers to mosquito samples that were undifferentiated between An. coluzzii and An. gambiae, or M and S forms, before they were recognised as two species. Non-significant results are denoted n.s.

Figure S6. Comparisons between resistance to etofenprox and five other pyrethroids, measured using standard WHO susceptibility tests in (**A**) the *An. gambiae* complex and (**B**) the *An. funestus* subgroup.

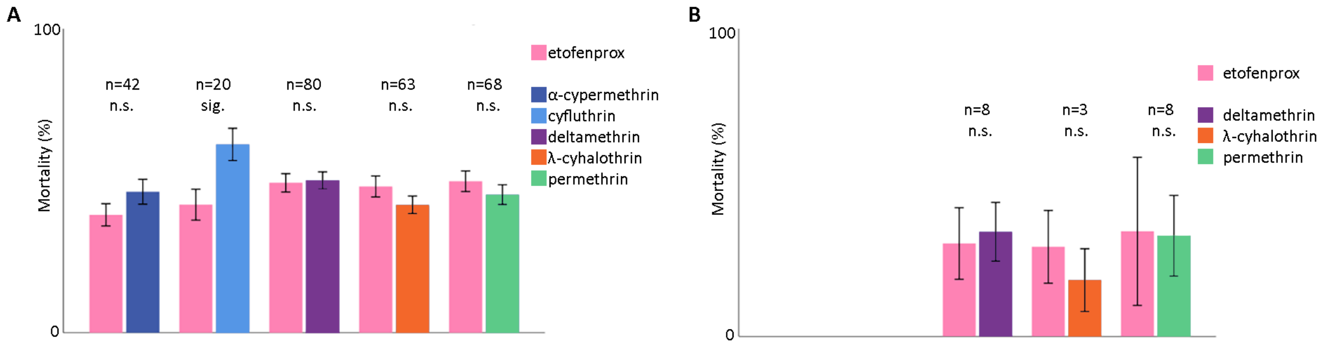

Significant (at the 0.05 level with a Holm-Bonferroni correction) results from a paired-sample t-test are denoted 'sig.', non-significant results are denoted 'n.s.', and 'n' is the number of pairs in each set.

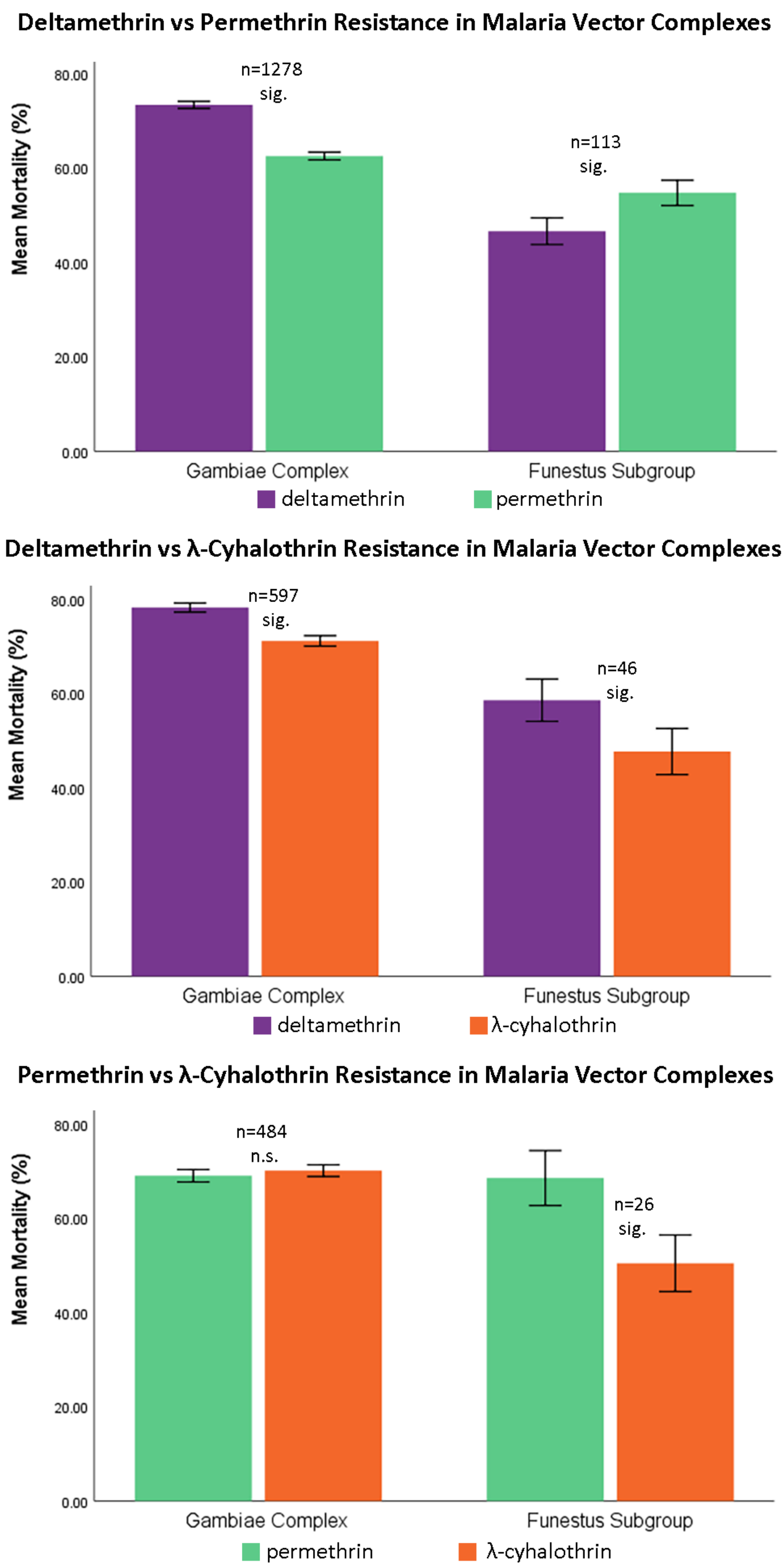

Figure S7. Comparison of resistance to three pyrethroids in the *An. gambiae* complex and *An. funestus* subgroup. Significant results (at the 0.05 level with a Holm-Bonferroni correction) from a paired-sample t-test are denoted ‘sig.’, non-significant results are denoted ‘n.s.’, and ‘n’ is the number of pairs in each set.

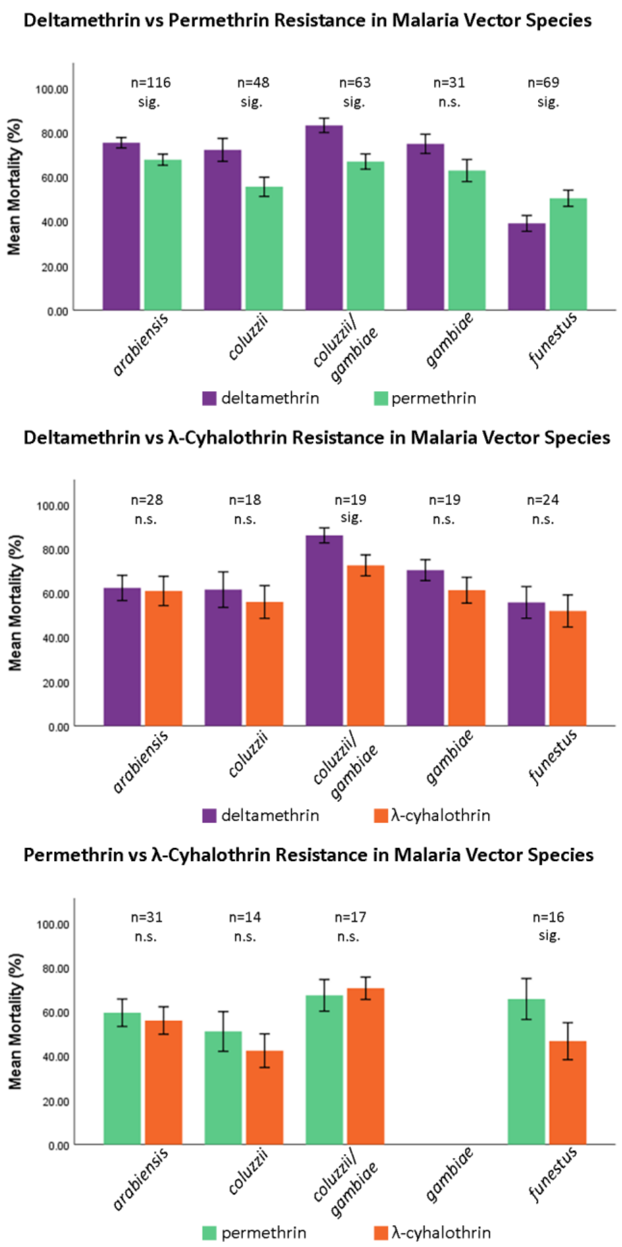

Figure S8. Comparison of resistance to three pyrethroids in four malaria vector species. Two species, *An. coluzzii* and *An. gambiae*, were previously classified as one and results for this former species are entitled 'coluzzii/gambiae'. Significant results (at the 0.05 level with a Holm-Bonferroni correction) from a paired-sample t-test are denoted 'sig.', non-significant results are denoted 'n.s.', and 'n' is the number of pairs in each set.

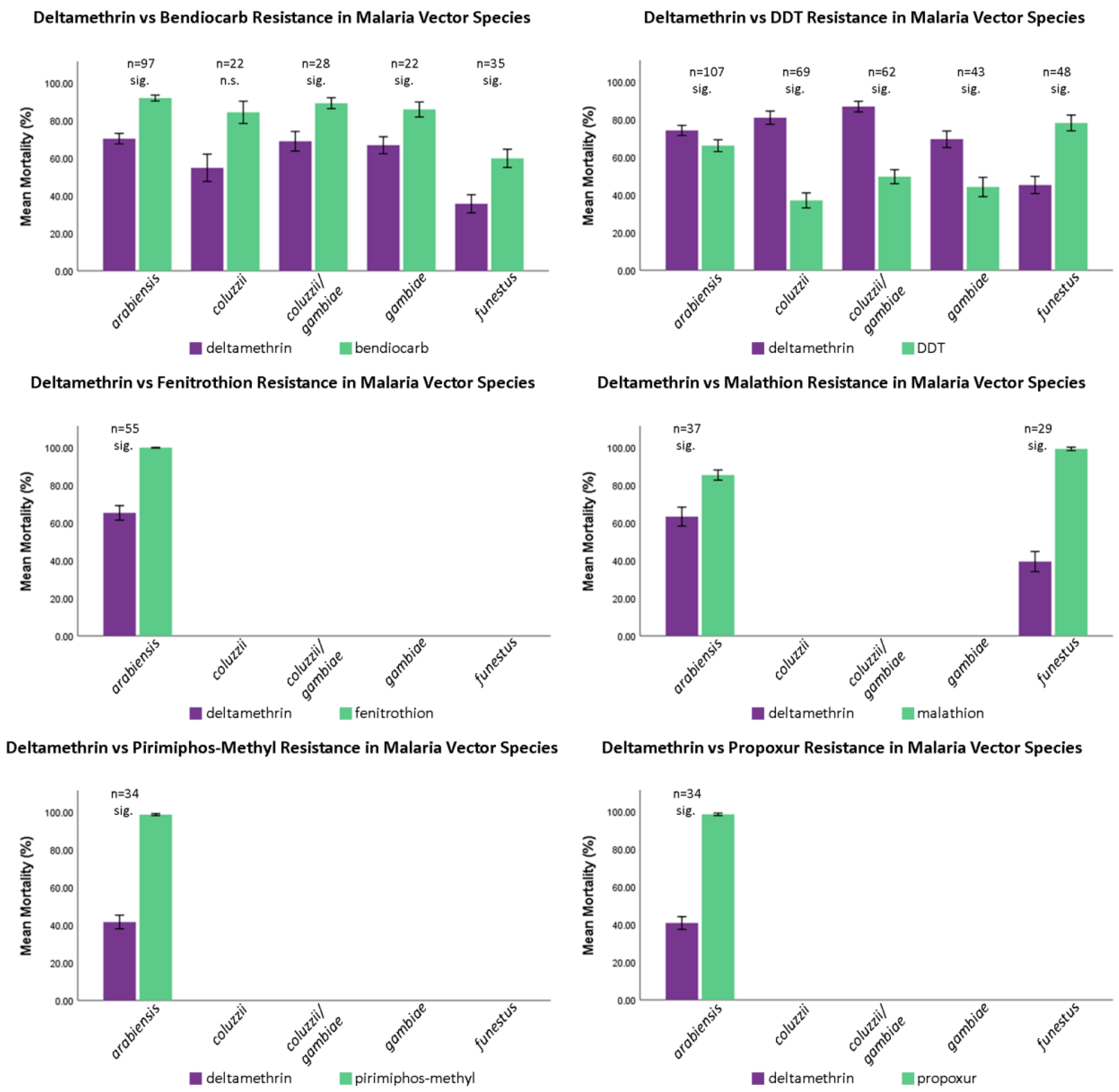

Figure S9. Comparison of resistance to deltamethrin and six insecticides from other classes. Two species, *An. coluzzii* and *An. gambiae*, were previously classified as one and results for this former species are entitled 'coluzzii/gambiae'. Significant results (at the 0.05 level with a Holm-Bonferroni correction) from a paired-sample t-test are denoted 'sig.', non-significant results are denoted 'n.s.', and 'n' is the number of pairs in each set.
