# Supplementary material for: Assessing cross-resistance within the pyrethroids in terms of their interactions with key cytochrome P450 enzymes and resistance in vector populations
Source: Parasit Vectors. 2021 Feb 18;14:115. doi: 10.1186/s13071-021-04609-5 (PMC7893915; doi:10.1186/s13071-021-04609-5)
Supplement: Supplementary file 3 — Additional file 3. Further details of the analyses of resistance in Aedes populations including Table S5. Diagnostic doses and data volumes for each pyrethroid. Table S6. Correlations in resistance to different pyrethroids in Ae. aegypti samples. Table S7. Comparisons of mean mortality between pairs of pyrethroids. [file 13071_2021_4609_MOESM3_ESM.pdf]

Additional File 3: Cross-resistance to pyrethroids within wild populations of *Aedes* vectors

Introduction

If structural diversity within the pyrethroids is also associated with divergence in resistance within the *Aedes* vectors of arboviruses, this would lend weight to the findings for malaria vectors presented in the main manuscript. Like the *Anopheles* vectors, target-site mutations and metabolic resistance are thought to be the main resistance mechanisms in *Aedes* mosquitoes [1-4]. At least ten mutations in the *Aedes aegypti Vgsc* gene have been identified and the most widespread mutation, 1534C, confers cross-resistance to permethrin and deltamethrin when in combination with other mutations and is also associated with DDT resistance [5, 6]. More recently, the V1016G mutation has been found in *Ae. albopictus* in Viet Nam and Italy, and both this allele and the F1534C and F1534S alleles are associated with resistance to permethrin, etofenprox and deltamethrin [7]. Over-expression of cytochrome P450s has also been implicated in pyrethroid resistance within *Aedes* mosquitoes and there is evidence that metabolic resistance can confer cross-resistance to multiple pyrethroids. For example, over-expression of CYP9J28 is associated with metabolic resistance to both permethrin and deltamethrin in *Ae. aegypti* [8] and when six P450 genes from *Ae. aegypti* were expressed in *Escherichia coli*, four metabolised both permethrin and deltamethrin [9]. A study of target site- and P450-mediated resistance in *Ae. aegypti* strains found variation in resistance to seven pyrethroids and hypothesised that some patterns of resistance could be linked to structural differences, but also noted that general conclusions are challenging to make without a greater number of compounds with a single modification to compare [3]. No studies of structure activity relationships using a wide range of pyrethroids and P450s from *Aedes* mosquitoes have been conducted to-date, but data on resistance in wild populations are available [1]. A wider range of pyrethroids are more typically tested by studies of resistance in *Aedes* vectors, compared to *Anopheles* vectors, although these studies are also more varied in terms of diagnostic dose, bioassay method and life cycle stage, making comparisons across studies difficult. Here we selected studies that used a common experimental design and investigated correlations in resistance to pairs of pyrethroids mirroring part of the work on *Anopheles* vectors presented in the main manuscript.

Methods

We identified all instances within a public insecticide resistance database [36] where an *Aedes* field sample had been subdivided among tests for different pyrethroids. We then identified the most commonly used bioassay type across the new pyrethroid dataset, which was the WHO adult susceptibility test as described for malaria vectors, and the most commonly used diagnostic dose for each compound (Table S2). Any results that did not use these doses or this bioassay type were removed. Paired results were then extracted, and the mean values for the Pearson's correlation coefficient were calculated for each pyrethroid pair using 1,000 bootstaps in SPSS Statistics v25.

Table S5. Diagnostic doses and data volumes for each pyrethroid.

Pyrethroid	Most common dose	Number of data points
α-cypermethrin	0.05	4
cyfluthrin	0.15	100
deltamethrin	0.05	177
etofenprox	0.5	32
λ-cyhalothrin	0.05	90
permethrin	0.75	168

The number of data points available for each pyrethroid from an *Ae. aegypti* sample that was used to test at least one other pyrethroid, using standard WHO susceptibility tests and standard diagnostic doses, is given.

Results

Resistance to cyfluthrin, deltamethrin, λ-cyhalothrin and permethrin was significantly correlated, whereas there were no significant correlations between these four pyrethroids and etofenprox

(Table S3). The pyrethroid α-cypermethrin was rarely tested at the same time as other pyrethroids using a standard diagnostic dose and standard adult susceptibility tests so no analyses involving this compound could be conducted. There were insufficient data and insufficient variation (mortality was typically equal to or close to 100%) for *Ae*. *albopictus* to repeat the analysis for this species.

Table S6. Correlations in resistance to different pyrethroids in *Ae. aegypti* samples

	N	R
deltamethrin vs cyfluthrin	72	0.850*
deltamethrin vs λ-cyhalothrin	67	0.684*
permethrin vs λ-cyhalothrin	56	0.644*
permethrin vs cyfluthrin	79	0.573*
deltamethrin vs permethrin	136	0.557*
λ-cyhalothrin vs cyfluthrin	25	0.553*
deltamethrin vs etofenprox	29	0.264
λ-cyhalothrin vs etofenprox	21	0.256
permethrin vs etofenprox	31	0.139
cyfluthrin vs etofenprox	22	0.045

Results are ranked with the most closely correlated pair at the top. Significant results (at the 0.05 level with a Holm-Bonferroni correction) are denoted by *.

The caveats about comparing the prevalence of resistance in *Anopheles* across different pyrethroids are even more important for studies of *Aedes* vectors because the diagnostic doses most commonly used for *Aedes* testing were taken from the guidance for *Anopheles* testing and were not calibrated for these *Aedes* species. When we compared these mortality values using paired sample t-tests, mortality was significantly lower following etofenprox exposure compared to deltamethrin, permethrin, λ-cyhalothrin and cyfluthrin exposure, with differences between the mean mortality values ranging from 42-67%. These substantial differences may reflect either calibration issues or genuinely higher resistance prevalence of to etofenprox in *Ae. aegypti* as predicted by the SAR studies of anopheline P450s.

Discussion

We have shown that resistance to different pyrethroids in *Ae aegypti* populations is typically correlated, but this is not true for etofenprox. This finding is in agreement with those for *Anopheles* vectors where resistance to etofenprox was less closely correlated to that for the other pyrethroids commonly used. In the case of *Ae. aegypti* populations, there were no significant correlations with etofenprox resistance at all, although the data volumes for etofenprox resistance in *Ae aegypti* were lower than those available for *An. gambiae* s.l. and a larger study may reveal weak but significant correlations.

Previous studies have shown that although resistance can vary among pyrethroids, the mechanisms identified to-date typically confer a degree of resistance to all of the pyrethroids tested. In two lab strains with i) target site- and P450-mediated resistance and ii) P450-mediated resistance alone, there was a positive resistance ratio for all seven pyrethroids tested (bioallethrin, permethrin, cyfluthrin, cypermethrin, fenpropathrin, etofenprox and (1R)-trans-fenfluthrin) whereas both strains were susceptible to two of the four organophosphates tested [3]. A study of an *Ae. aegypti* strain from Puerto Rico with both target site- and P450-mediated resistance found resistance to three pyrethroids (permethrin, -cypermethrin, etofenprox) as well as to three other compounds that interact with the sodium channel whereas this strain was susceptible to the pyrrole, chlorfenapyr, that acts as a mitochondrial electron transport inhibitor [10]. A study of a resistant *Ae. aegypti* strain from Madeira found resistance to each of cyfluthrin, permethrin and fenitrothion was associated with P450-mediated resistance and the same was true for the carbamate, bendiocarb [11].

Our structure activity findings using P450s from *Anopheles* mosquitoes indicated resistance to bifenthrin could diverge from that to the other pyrethroids tested. Bifenthrin wasn't included in the studies that met the inclusion criteria for our analysis of resistance in *Aedes* populations, but has investigated by other studies of resistance in Aedes populations. One study in Mexico tested seven populations of *Aedes aegypti* with eight pyrethroids and compared the concentrations required for 50% knockdown (KC_{50}) and mortality (LC_{50}) to the same values obtained using a susceptible strain to give a resistance ratio (RR) [12]. Across the seven populations, resistance to deltamethrin, lambda-cyhalothrin, permethrin and α-cypermethrin were highly correlated (in terms of both $RRKC_{50}$ and $RRLC_{50}$), indicating the existence of strong cross-resistance. However, the resistance values for bifenthrin were not correlated with any of those for the other four compounds and the study concluded bifenthrin could be used as an alternative insecticide for *Ae. aegypti* control in Mexico. Two independent studies in Thailand tested three *Ae. aegypti* and three *Ae. albopictus* populations, respectively, and calculated the diagnostic doses for each pyrethroid including bifenthrin using a susceptible strain [13, 14]. In both instances, the population with the highest deltamethrin resistance also had the highest bifenthrin resistance, so no evidence for divergence in resistance was observed for these two species in Thailand. Given the known data noise in susceptibility test results, caution is needed when interpreting the results from a single study at a small number of sites.

Control of diurnally-active, outdoors-biting *Aedes* vectors is not focused on insecticide-treated bed nets in the way it is for the control of African malaria vectors, however, pyrethroids including bioresmethrin, cyfluthrin, cypermethrin, cyphenothrin, D-phenothrin, etofenprox, λ-cyhalothrin, permethrin and resmethrin are deployed in sprays, ovitraps, and materials such as window curtains [15, 16]. This means that questions about switching between pyrethroids may still arise. Here we have shown correlations between resistance in Ae. aegypti populations to cyfluthrin, deltamethrin, λ-cyhalothrin and permethrin. That is a populations with higher resistance to one of these pyrethroids is likely to have higher resistance to the others, so it would be inadvisable to switch between them.

Table S7. Comparisons of mean mortality between pairs of pyrethroids.

Pair	N	Pyrethroid	Mean percent mortality (SE)	Difference in percent mortality
1	29	deltamethrin	90.68 (2.80)	69.09*
		etofenprox	21.59 (4.75)	
2	22	cyfluthrin	87.78 (3.56)	66.64*
		etofenprox	21.14 (5.32)	
3	21	λ-cyhalothrin	72.40 (4.52)	55.59*
		etofenprox	16.81 (4.43)	
4	31	permethrin	63.39 (5.36)	42.30*
		etofenprox	21.09 (4.89)	
5	79	permethrin	59.01 (3.18)	30.25*
		cyfluthrin	89.27 (1.61)	
6	136	deltamethrin	88.10 (1.66)	26.66*
		permethrin	61.44 (2.40)	
7	25	λ-cyhalothrin	66.90 (5.01)	18.11*
		cyfluthrin	85.01 (3.81)	
8	56	permethrin	70.10 (3.99)	14.42*
		λ-cyhalothrin	84.52 (3.40)	
9	67	deltamethrin	85.64 (2.66)	11.49*
		λ-cyhalothrin	74.16 (3.55)	
10	72	deltamethrin	88.43 (1.94)	$0.15^{n.s.}$
		cyfluthrin	88.58 (1.73)	

* denotes a significant difference between the percent mortality values for two pyrethroids at the 0.05 level with a Holm-Bonferroni correction. Non-significant results are denoted n.s.

References

1. Moyes CL, Vontas J, Martins AJ, Ng LC, Koou SY, Dusfour I, et al. Contemporary status of insecticide resistance in the major *Aedes* vectors of arboviruses infecting humans. Plos Neglected Tropical Diseases. 2017;11 7; doi: 10.1371/journal.pntd.0005625.
2. Samantsidis GR, Panteleri R, Denecke S, Kounadi S, Christou I, Nauen R, et al. 'What I cannot create, I do not understand': functionally validated synergism of metabolic and target site insecticide resistance. Proceedings of the Royal Society B-Biological Sciences. 2020;287 1927; doi: 10.1098/rspb.2020.0838.
3. Smith LB, Sears C, Sun H, Mertz RW, Kasai S, Scott JG. CYP-mediated resistance and cross-resistance to pyrethroids and organophosphates in Aedes aegypti in the presence and absence of kdr. Pesticide Biochemistry and Physiology. 2019;160:119-26; doi: 10.1016/j.pestbp.2019.07.011.
4. Saavedra-Rodriguez K, Campbell CL, Lenhart A, Penilla P, Lozano-Fuentes S, Black WC. Exome-wide association of deltamethrin resistance in Aedes aegypti from Mexico. Insect Molecular Biology. 2019;28 5:591-604; doi: 10.1111/imb.12575.
5. Harris AF, Rajatileka S, Ranson H. Pyrethroid Resistance in Aedes aegypti from Grand Cayman. American Journal of Tropical Medicine and Hygiene. 2010;83 2:277-84; doi: 10.4269/ajtmh.2010.09-0623.
6. Kushwah RBS, Dykes CL, Kapoor N, Adak T, Singh OP. Pyrethroid-Resistance and Presence of Two Knockdown Resistance (kdr) Mutations, F1534C and a Novel Mutation T1520I, in Indian Aedes aegypti. Plos Neglected Tropical Diseases. 2015;9 1; doi: 10.1371/journal.pntd.0003332.
7. Kasai S, Caputo B, Tsunoda T, Cuong TC, Maekawa Y, Lam-Phua SG, et al. First detection of a Vssc allele V1016G conferring a high level of insecticide resistance in Aedes albopictus collected from Europe (Italy) and Asia (Vietnam), 2016: a new emerging threat to controlling arboviral diseases. Eurosurveillance. 2019;24 5:48-59; doi: 10.2807/1560-7917.Es.2019.24.5.1700847.
8. Badolo A, Sombie A, Pignatelli PM, Sanon A, Yameogo F, Wangrawa DW, et al. Insecticide resistance levels and mechanisms in Aedes aegypti populations in and around Ouagadougou, Burkina Faso. Plos Neglected Tropical Diseases. 2019;13 5; doi: 10.1371/journal.pntd.0007439.
9. Stevenson BJ, Pignatelli P, Nikou D, Paine MJI. Pinpointing P450s Associated with Pyrethroid Metabolism in the Dengue Vector, Aedes aegypti: Developing New Tools to Combat Insecticide Resistance. Plos Neglected Tropical Diseases. 2012;6 3; doi: 10.1371/journal.pntd.0001595.
10. Estep AS, Sanscrainte ND, Waits CM, Louton JE, Becnel JJ. Resistance Status and Resistance Mechanisms in a Strain of Aedes aegypti (Diptera: Culicidae) From Puerto Rico. Journal of Medical Entomology. 2017;54 6:1643-8; doi: 10.1093/jme/tjx143.
11. Seixas G, Grigoraki L, Weetman D, Vicente JL, Silva AC, Pinto J, et al. Insecticide resistance is mediated by multiple mechanisms in recently introduced Aedes aegypti from Madeira Island (Portugal). Plos Neglected Tropical Diseases. 2017;11 7; doi: 10.1371/journal.pntd.0005799.
12. Flores AE, Ponce G, Silva BG, Gutierrez SM, Bobadilla C, Lopez B, et al. Wide spread cross resistance to pyrethroids in *Aedes aegypti* (Diptera: Culicidae) from Veracruz State Mexico. Journal of Economic Entomology. 2013;106 2:959-69; doi: 10.1603/ec12284.
13. Juntarajumnong W, Pimnon S, Bangs MJ, Thanispong K, Chareonviriyaphap T. Discriminating lethal concentrations and efficacy of six pyrethroids for control of *Aedes aegypti* in Thailand. Journal of the American Mosquito Control Association. 2012;28 1:30-7; doi: 10.2987/11-6203.1.
14. Thanispong K, Sathantriphop S, Malaithong N, Bangs MJ, Chareonviriyaphap T. Establishment of diagnostic doses of five pyrethroids for monitoring physiological resistance

in *Aedes albopictus* in Thailand. Journal of the American Mosquito Control Association. 2015;31 4:346-52

15. Morrison AC, Zielinski-Gutierrez E, Scott TW, Rosenberg R. Defining challenges and proposing solutions for control of the virus vector Aedes aegypti. Plos Medicine. 2008;5 3:362-6; doi: 10.1371/journal.pmed.0050068.
16. World Health Organization: Dengue: Guidelines for Diagnosis, Treatment, Prevention and Control. Geneva, Switzerland2009: 160.
